# Supplementary material for: Exotic plants accumulate and share herbivores yet dominate communities via rapid growth
Source: Nat Commun. 2021 May 11;12:2696. doi: 10.1038/s41467-021-23030-1 (PMC8113582; doi:10.1038/s41467-021-23030-1)
Supplement: Supplementary file 1 — Supplementary Information [file 41467_2021_23030_MOESM1_ESM.pdf]

Supplementary Information for Allen et al., Exotic plants accumulate and share herbivores yet dominate communities via rapid growth

Warwick J. Allen[1,2]*, Lauren P. Waller[1,2], Barbara I. P. Barratt[3,4], Ian A. Dickie[1†], Jason M. Tylianakis[1†]

[1] The Bio-Protection Research Centre, School of Biological Sciences, University of Canterbury, Christchurch 8041, New Zealand

[2] The Bio-Protection Research Centre, Lincoln University, Lincoln 7647, New Zealand

[3] AgResearch, Invermay Research Centre, Mosgiel 9053, New Zealand

[4] Department of Botany, University of Otago, PO Box 56, Dunedin, New Zealand

* Corresponding author: warwick.j.allen@gmail.com; +642041251085; School of Biological Sciences, University of Canterbury, Private Bag 4800, Christchurch 8041, New Zealand

[†] Joint senior authors

Supplementary Figures

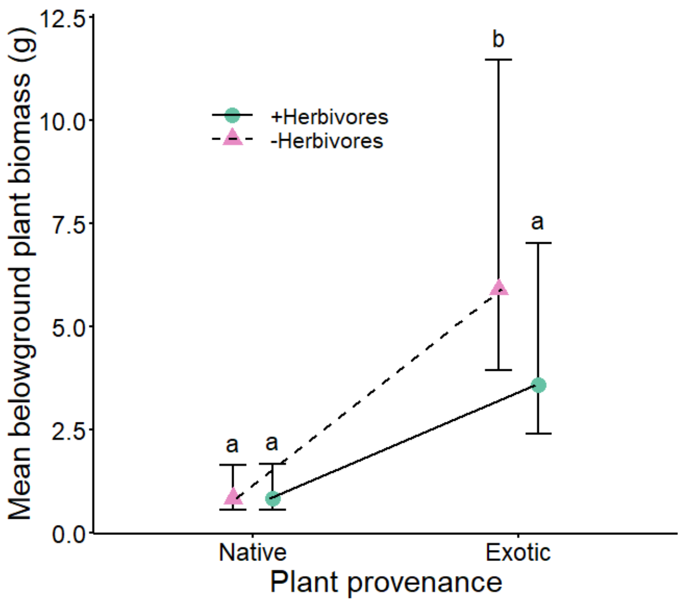

Supplementary Figure 1. Influence of the herbivore treatment on belowground biomass of native and exotic plants. Exotic plants (n = 211 and 206 in -Herbivore and +Herbivore mesocosms, respectively) produced 39% less belowground biomass in +Herbivore (green circles and solid line) compared with -Herbivore (pink triangles and dashed line) mesocosms (P = 0.002, Bonferroni corrected pairwise Tukey test based on the plant provenance × herbivore treatment interaction: $F_{1,883}$ = 8.25, P = 0.004, whereas the herbivore treatment did not affect native plants (P = 1.000, n = 273 and 261 in -Herbivore and +Herbivore mesocosms, respectively). Exotic plants produced 7 times more belowground biomass than natives when herbivores were absent (P = 0.029). Different lowercase letters indicate significant differences (P < 0.05, based on Bonferroni corrected Tukey tests) between back-transformed estimated marginal means (± SEM) from the linear mixed model. Corresponding violin plots showing the distribution of raw data are presented in Supplementary Fig. 18.

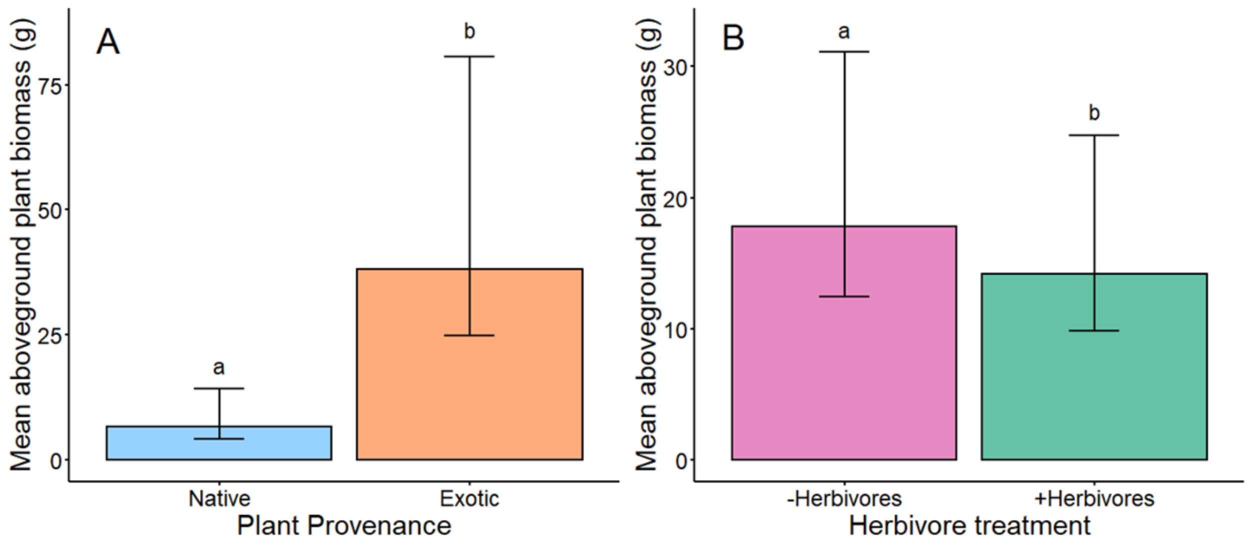

Supplementary Figure 2. Influence of plant provenance and the herbivore addition treatment on aboveground plant biomass. A) Exotic plant species (orange, n = 417) produced 5.8 times higher aboveground plant biomass than natives (blue, n = 534) ($F_{1,36}$ = 5.52, *P* = 0.024). B) plants subjected to the added (green, n = 467) rather than the reduced (pink, n = 484) herbivore treatment produced 20% less aboveground biomass ($F_{1,884}$ = 7.56, *P* = 0.006). Different lowercase letters indicate significant differences (*P* < 0.05) between back-transformed estimated marginal means (± SEM) from the linear mixed model. Corresponding violin plots showing the distribution of raw data are presented in Supplementary Fig. 19.

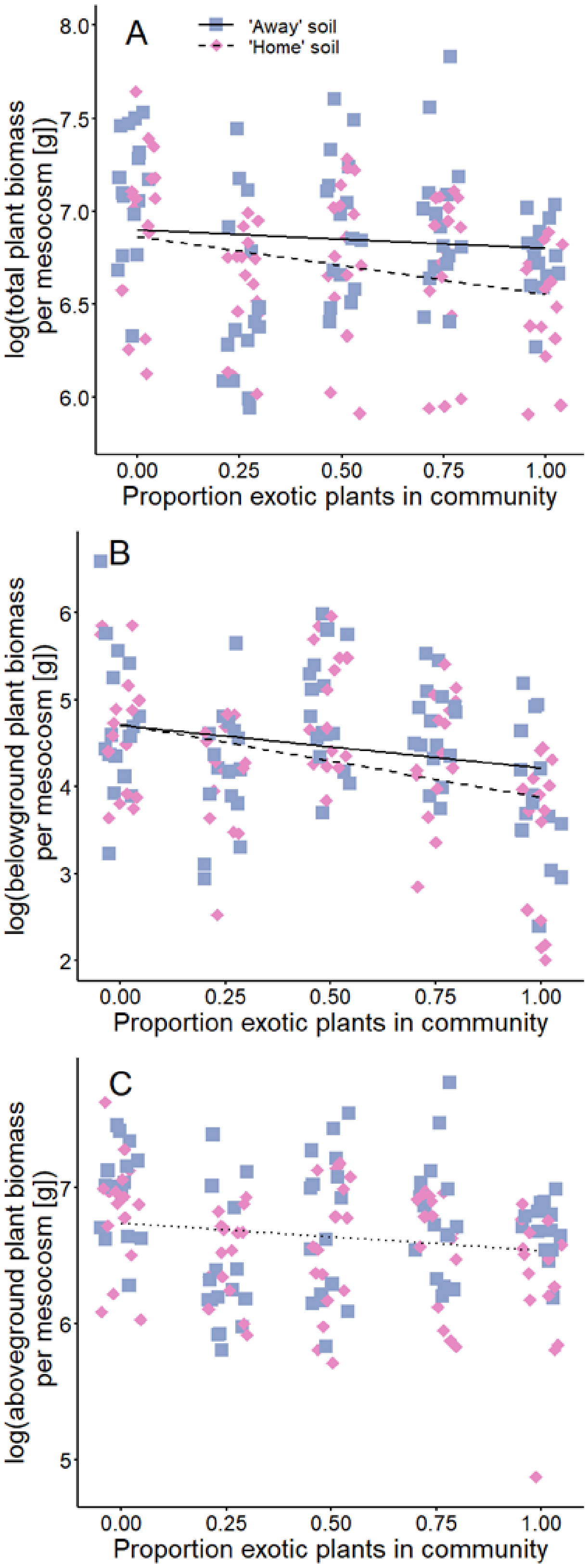

Supplementary Figure 3. Influence of the proportion of exotic plant species and soil treatment on plant biomass. A) Total plant biomass of mesocosms (log-transformed) did not vary with the proportion of exotics planted for either soil treatment ('home' = pink

diamonds and dashed line: slope = -0.38, t = -1.29, P = 0.198; ‘away’ = purple squares and solid line: slope = -0.12, t = -0.53, P = 0.600; n = 80 mesocosms per soil treatment), although the slope of the two relationships differed from one another (proportion of exotic plants × soil treatment interaction: $F_{1,134}$ = 4.27, P = 0.041). B) Belowground plant biomass of mesocosms (log-transformed) decreased with the proportion of exotics planted in the ‘home’ soil treatment (slope = -1.21, t = -2.44, P = 0.021) but not in ‘away’ soil (slope = -0.65, t = -1.31, P = 0.200; proportion of exotic plants × soil treatment interaction: $F_{1,134}$ = 4.93, P = 0.028; n = 80 mesocosms per soil treatment). C) Aboveground plant biomass of mesocosms (log-transformed; n = 80 mesocosms per soil treatment) did not vary with the proportion of exotics planted (dotted line; $F_{1,18}$ = 0.94, P = 0.345) or differ between soil treatments ($F_{1,134}$ = 0.01, P = 0.942). A small amount of jitter has been added to separate overlapping points on the x-axis.

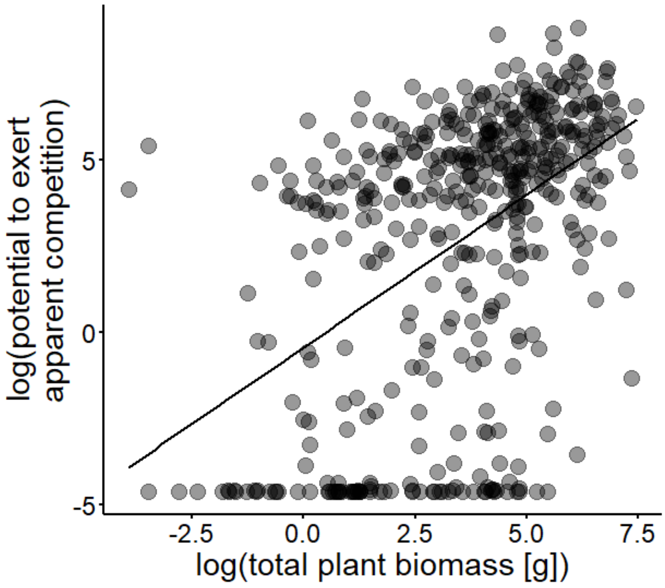

Supplementary Figure 4. Relationship between plant biomass and potential to exert apparent competition. Plants (n = 467) with higher biomass exhibited stronger potential to exert apparent competition ($PAC_{exerted}$) on the surrounding plant community via shared herbivores (slope = 0.004, $F_{1,437}$ = 23.74, P < 0.001). Both variables log-transformed to aid plotting. Darker shading indicates multiple overlapping datapoints.

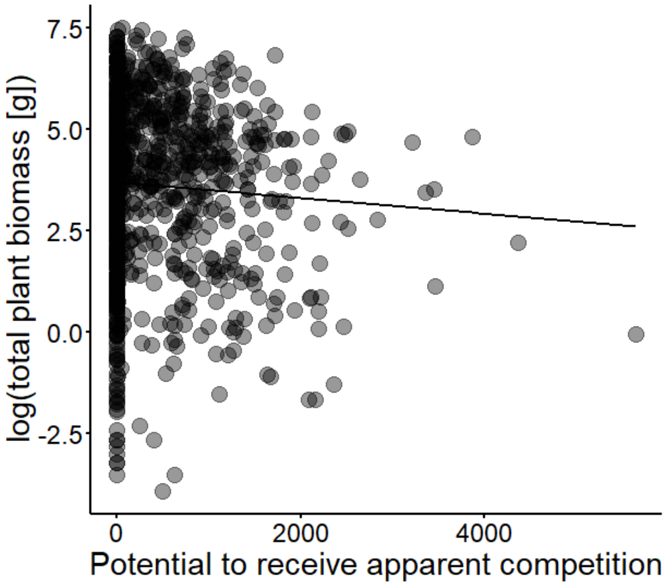

Supplementary Figure 5. Relationship between potential to receive apparent competition and plant biomass. Plants (n = 951) that produced lower total biomass (log-transformed) also experienced higher potential to receive apparent competition ($PAC_{received}$) from other plant species in the mesocosm community via shared herbivores (slope = -0.0007, $F_{1,899}$ = 5.26, P = 0.022). Darker shading indicates multiple overlapping datapoints.

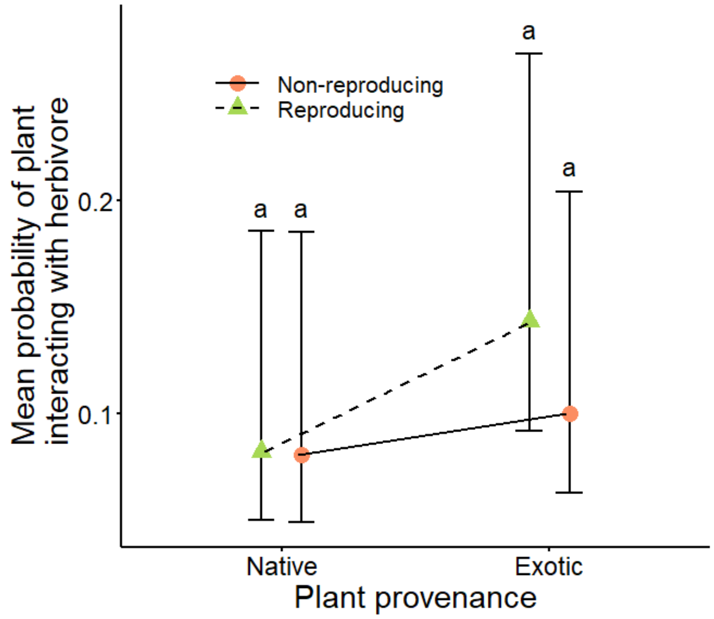

Supplementary Figure 6. Influence of herbivore reproductive status on the probability of it feeding on native and exotic plants. The probability of a herbivore species feeding on a given plant species within its fundamental host range depended on the interaction between plant provenance and insect reproduction status (reproducing = green triangles and dashed line; non-reproducing = orange circles and solid line) in the mesocosms (plant provenance × insect reproduction status interaction: $F = 6.00$, $P = 0.015$). However, identical lowercase letters for all treatment combinations indicate that there were no significant differences ($P < 0.05$, based on Bonferroni corrected Tukey tests) detected between back-transformed estimated marginal means (± SEM). For native plants, n = 986 and 1,684 potential interactions involving non-reproducing (308 realised interactions) and reproducing (338 realised) herbivores, respectively. For exotic plants, n = 976 and 1,900 potential interactions involving non-reproducing (392 realised interactions) and reproducing (569 realised) herbivores, respectively.

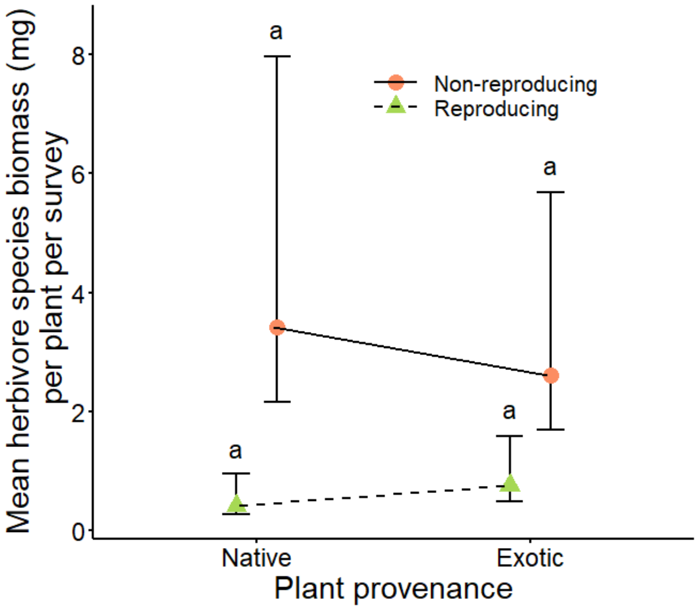

Supplementary Figure 7. Influence of herbivore reproductive status on herbivore species biomass on native and exotic plants. Herbivore species on plants depended on the interaction between plant provenance and insect reproduction status (reproducing = green triangles and dashed line; non-reproducing = orange circles and solid line) in the mesocosms (plant provenance × insect reproduction status interaction: $F_{1,1406} = 66.76$, $P = 6.8e^{-16}$). However, identical lowercase letters for all treatment combinations indicate that there were no significant differences ($P < 0.05$, based on Bonferroni corrected Tukey tests) detected between back-transformed estimated marginal means (± SEM). For native plants, n = 289 and 312 for non-reproducing and reproducing herbivores, respectively. For exotic plants, n = 337 and 517 for non-reproducing and reproducing herbivores, respectively. Corresponding violin plots showing the distribution of raw data are presented in Supplementary Fig. 20.

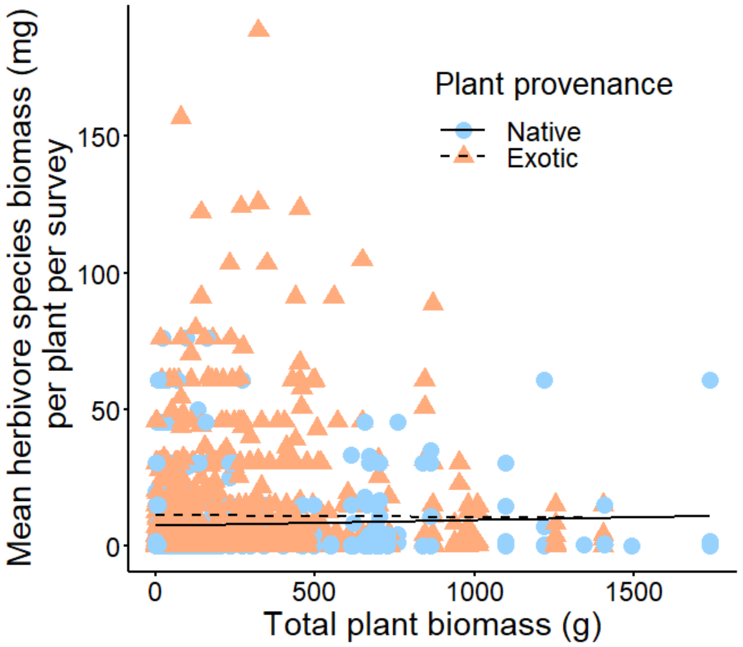

Supplementary Figure 8. Relationship between herbivore species biomass and total biomass of native and exotic plants. The relationship between herbivore species biomass and total plant biomass was positive for native (blue circles and solid line; slope = 0.0007, t = 3.47, P = 0.001, n = 601) but not exotic plants (orange triangles and dotted line; slope = 0.0002, t = 1.05, P = 0.295, n = 854; plant biomass × plant provenance interaction: $F_{1,382}$ = 4.10, P = 0.044).

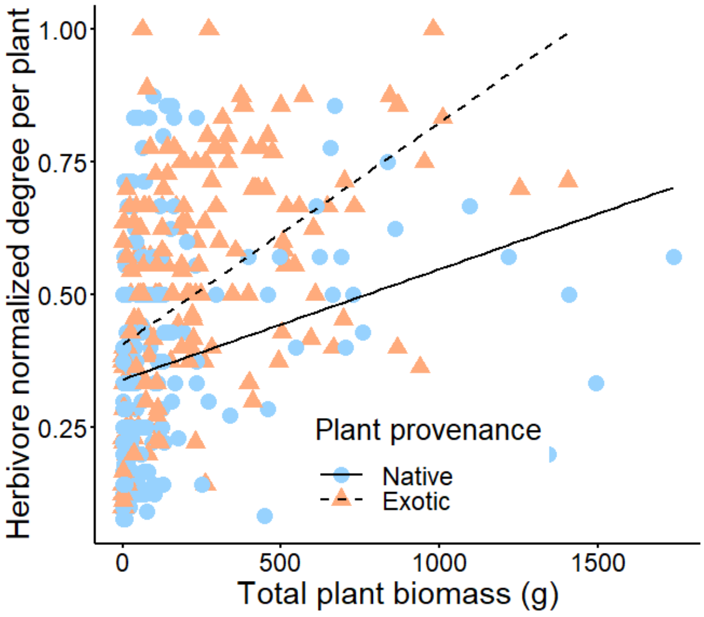

Supplementary Figure 9. Relationship between herbivore normalized degree and total biomass of native and exotic plants. The relationship between herbivore normalized degree (i.e., the proportion of herbivore species that fed on a given host plant out of the total herbivore species in the mesocosm) and total plant biomass was positive for exotic (orange triangles and dotted line; slope = 0.0003, t = 4.84, P = 0.000002, n = 259) but not native plants (blue circles and solid line; slope = 0.0001, t = 1.78, P = 0.076, n = 262; plant biomass × plant provenance interaction: $F_{1,348}$ = 5.01, P = 0.026).

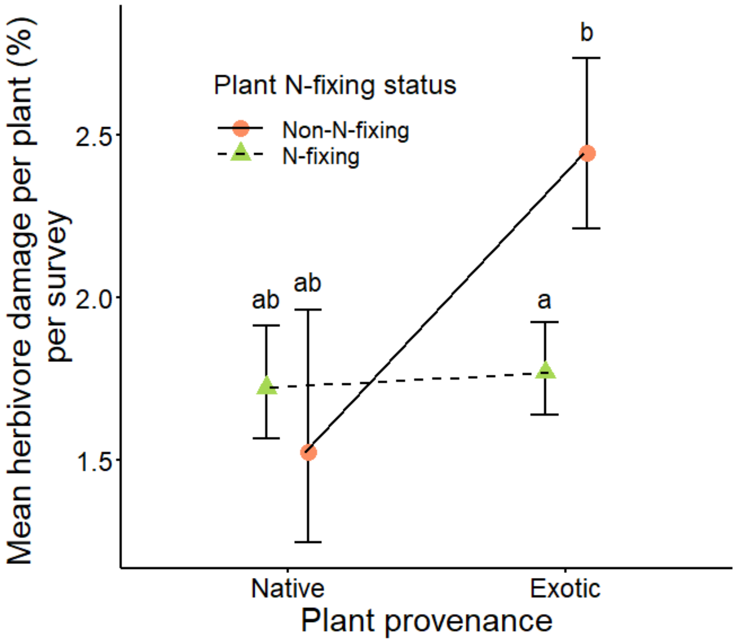

Supplementary Figure 10. Influence of plant nitrogen-fixing status on herbivore chewing and scraping damage to leaf tissue of native and exotic plants. Herbivore chewing and scraping damage was 38% higher on exotic plants that do not fix nitrogen compared with nitrogen-fixers (P = 0.022; n = 156 and 50 for non-N-fixing and N-fixing plants, respectively), whereas no difference was observed for native plants (n = 253 and 8 for non-N-fixing and N-fixing plants, respectively; nitrogen fixing status × plant provenance interaction: F = 8.96, P = 0.043). Different lowercase letters indicate significant differences (P < 0.05, based on Bonferroni corrected Tukey tests) between back-transformed estimated marginal means (± SEM). Corresponding violin plots showing the distribution of raw data are presented in Supplementary Fig. 21.

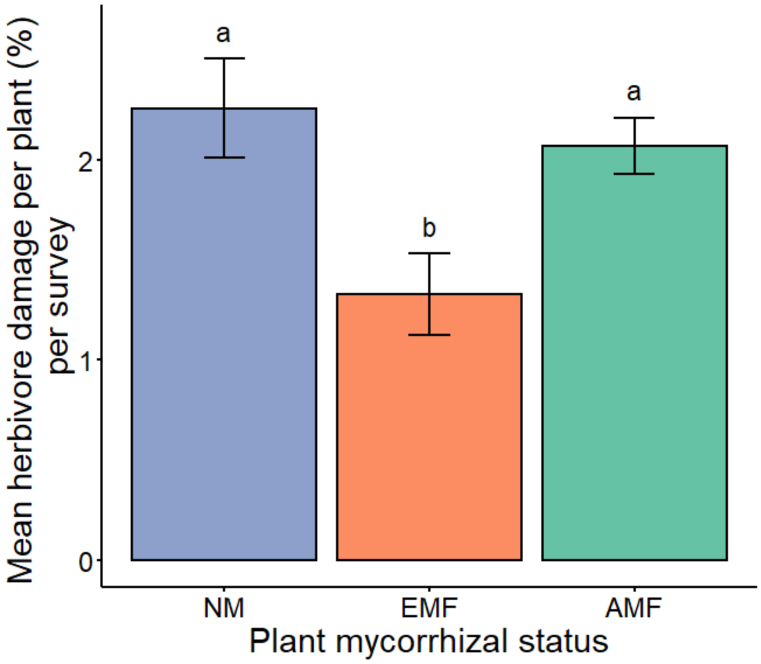

Supplementary Figure 11. Influence of plant mycorrhizal status on herbivore chewing and scraping damage to plant leaf tissue. Non-mycorrhizal plants (NM, blue, n = 104) and plants that associate with arbuscular mycorrhizal fungi (AMF, green, n = 334) respectively suffered 69% (P = 0.002) and 56% (P = 0.007) more herbivore chewing and scraping damage to leaf tissue than plants that associate with ectomycorrhizal fungi (EMF, orange, n = 29) (mycorrhizal status main effect: F = 25.90, P = 0.003). Different lowercase letters indicate significant differences (P < 0.05, based on Bonferroni corrected Tukey tests) between back-transformed estimated marginal means (± SEM). Corresponding violin plots showing the distribution of raw data are presented in Supplementary Fig. 22.

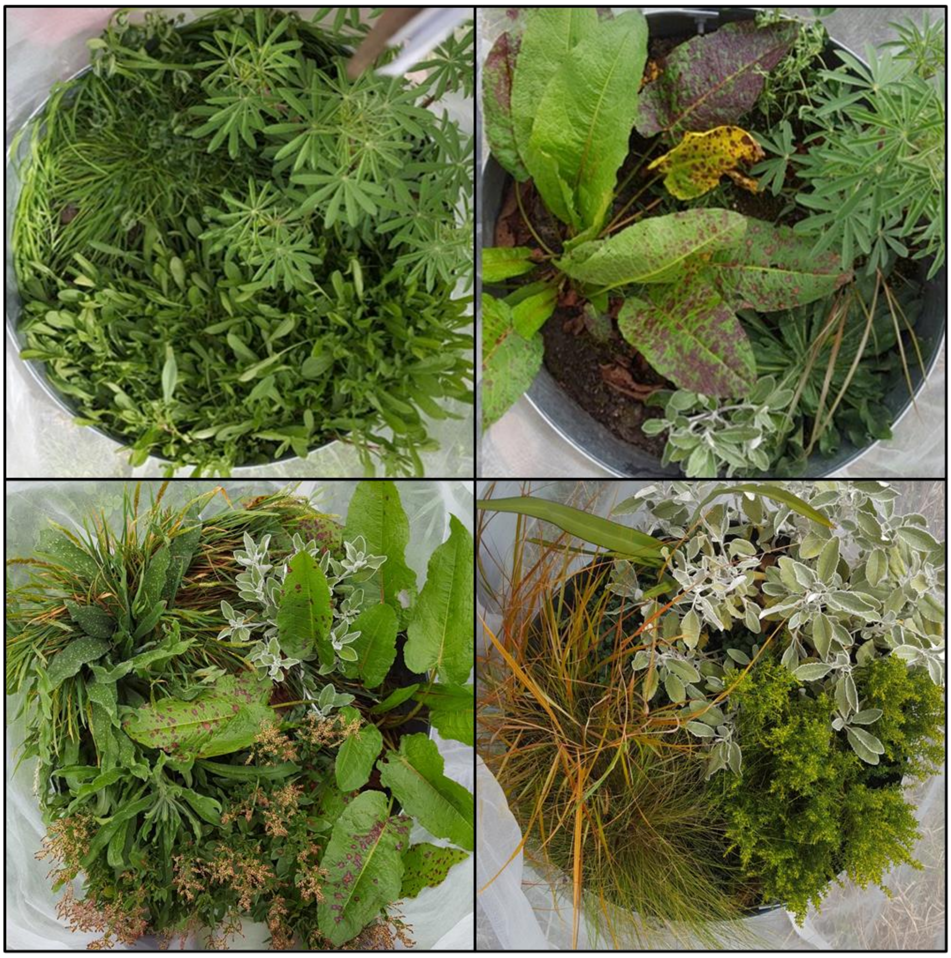

Supplementary Figure 12. Experimental mesocosm communities. Example experimental mesocosm communities after eight months of growth (community number, clockwise from top left: 16, 12, 5, 3).

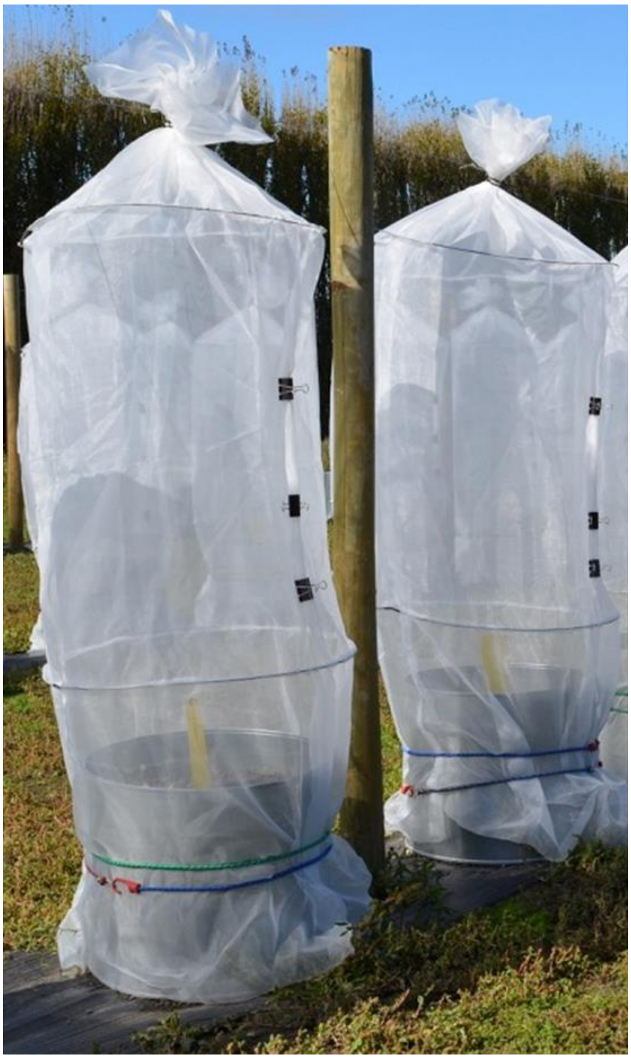

Supplementary Figure 13. Photo of herbivore cages. Herbivore cages installed on mesocosm pots prior to planting.

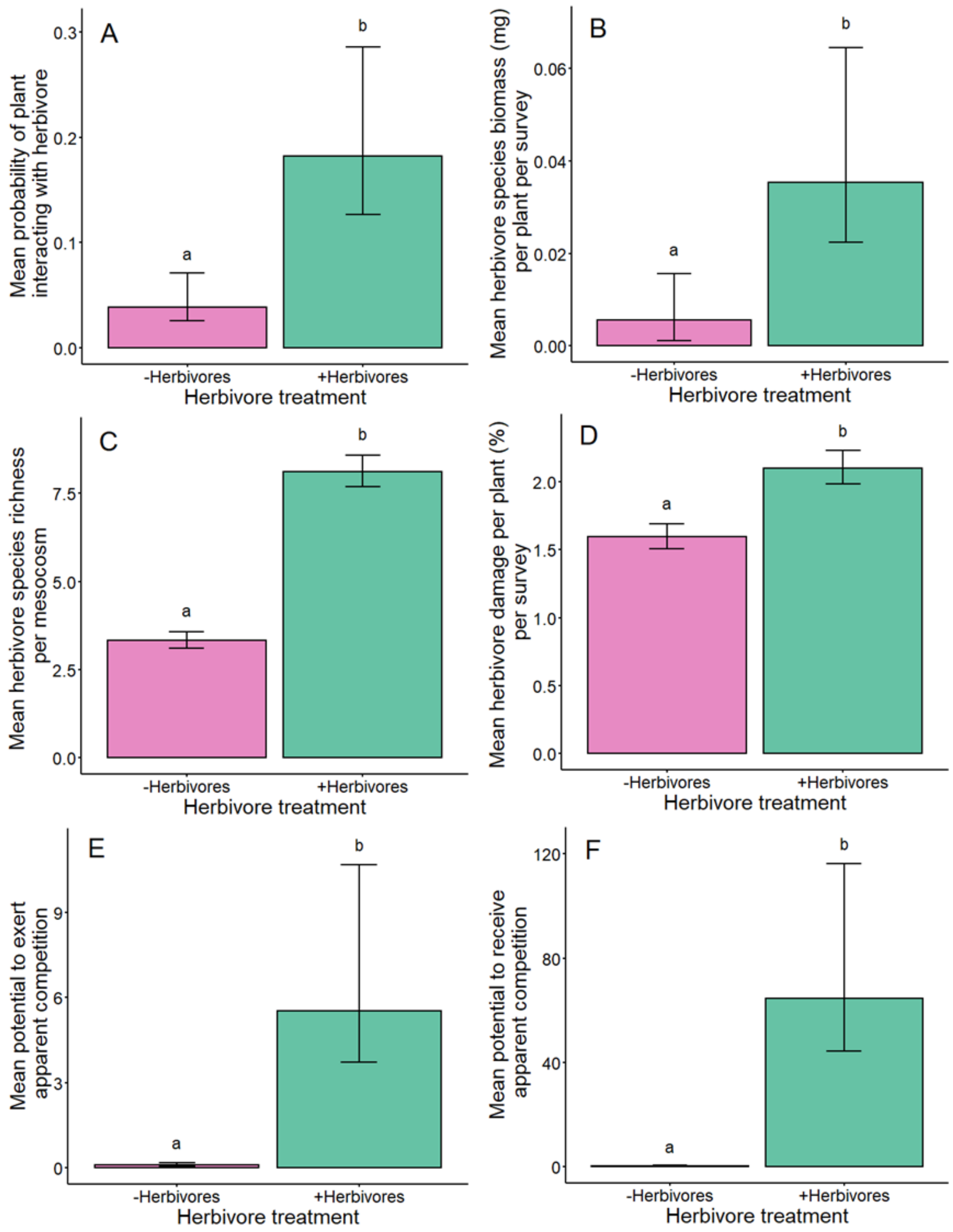

Supplementary Figure 14. Influence of the herbivore treatment on plant-herbivore interactions in experimental mesocosm communities. A) The herbivore exclusion treatment reduced A) herbivore species presence on plants within their fundamental host range by 79% ($F_{1,585}$ = 584.68, P < 0.001; n = 5,528 potential interactions each in -Herbivore

and +Herbivore mesocosms, with 535 and 1,607 realised, respectively), B) herbivore species biomass per plant per survey by 84% ($F_{1,137}$ = 651.55, P < 0.001; n = 5,528 potential interactions each in -Herbivore and +Herbivore mesocosms; absent interactions were included in this analysis because quantifying biomass of only species that were inside mesocosms does not test the effectiveness of the cage), C) herbivore species richness per mesocosm by 59% (F = 152.10, P < 0.001; n = 80 mesocosms each in -Herbivore and +Herbivore mesocosms), D) percent chewing and scraping damage to leaf tissue per plant by 24% (F = 276.22, P < 0.001; n = 640 each in -Herbivore and +Herbivore mesocosms), and the potential to E) exert and F) receive apparent competition with other plant species via shared herbivores by 98% ($F_{1,139}$ = 342.64, P < 0.001; n = 640 each in -Herbivore and +Herbivore mesocosms) and 99.5% ($F_{1,139}$ = 275.50, P < 0.001; n = 640 each in -Herbivore and +Herbivore mesocosms), respectively. Different lowercase letters indicate significant differences (P < 0.05) between back-transformed estimated marginal means (± SEM). Corresponding violin plots showing the distribution of raw data are presented in Supplementary Fig. 23.

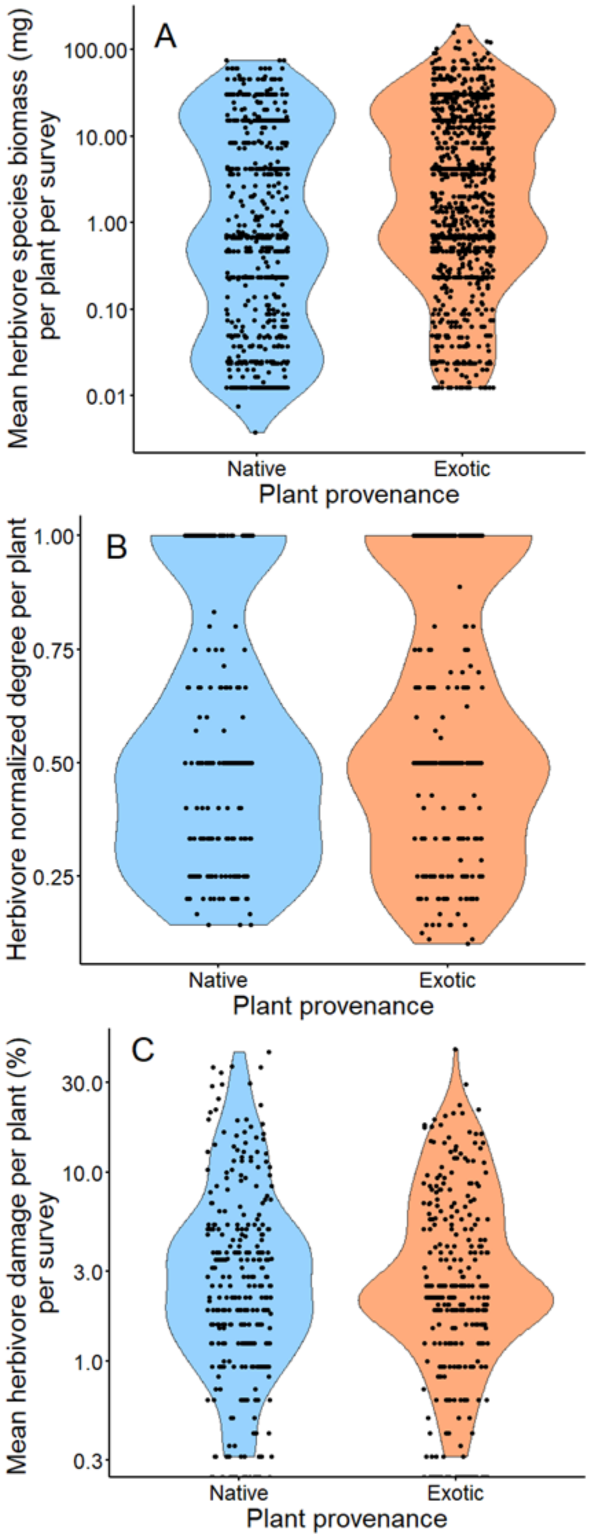

Supplementary Figure 15. Violin plots showing the distribution of raw data underlying the estimated marginal means presented in Fig. 2. A) Herbivore species biomass, B) normalized degree, and C) chewing and scraping damage on native (blue) and exotic (orange) plants. Data in panels A and C are plotted on a log-scale.

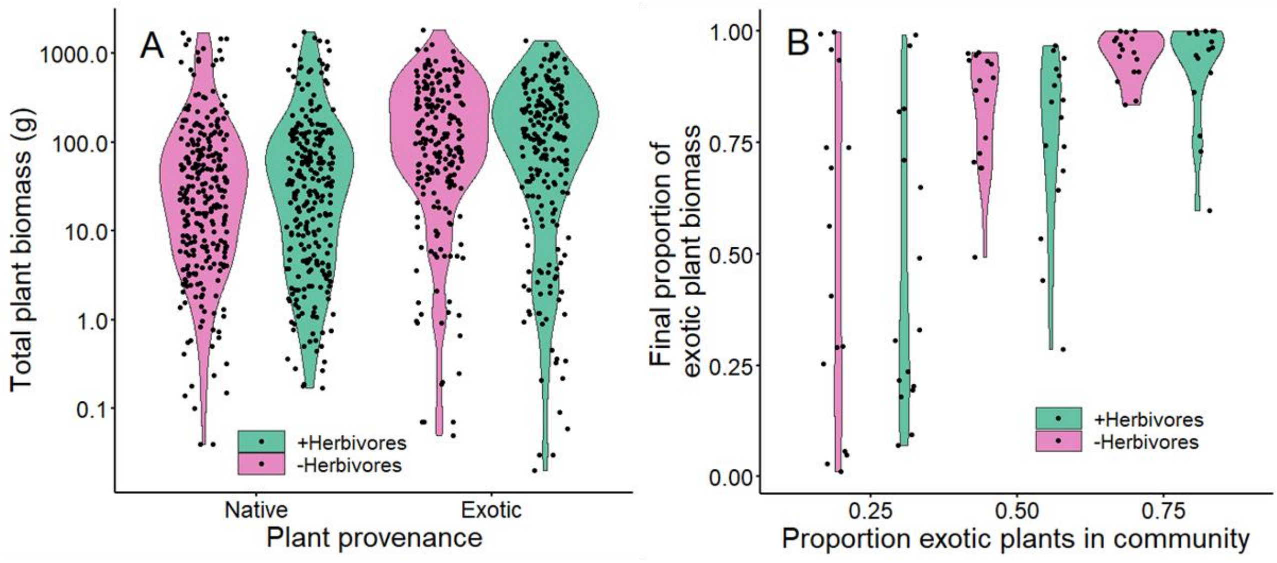

Supplementary Figure 16. Violin plots showing the distribution of raw data underlying the means presented in Fig. 3. A) Total plant biomass of native and exotic plants in mesocosms subjected to different herbivore treatments (pink = -Herbivores; green = +Herbivores). B) Proportion of total mesocosm biomass that was made up of exotic plants in mesocosms subjected to each herbivore treatment (pink = -Herbivores; green = +Herbivores), depending on proportion of exotics planted in the community. Data in panel A is plotted on a log-scale.

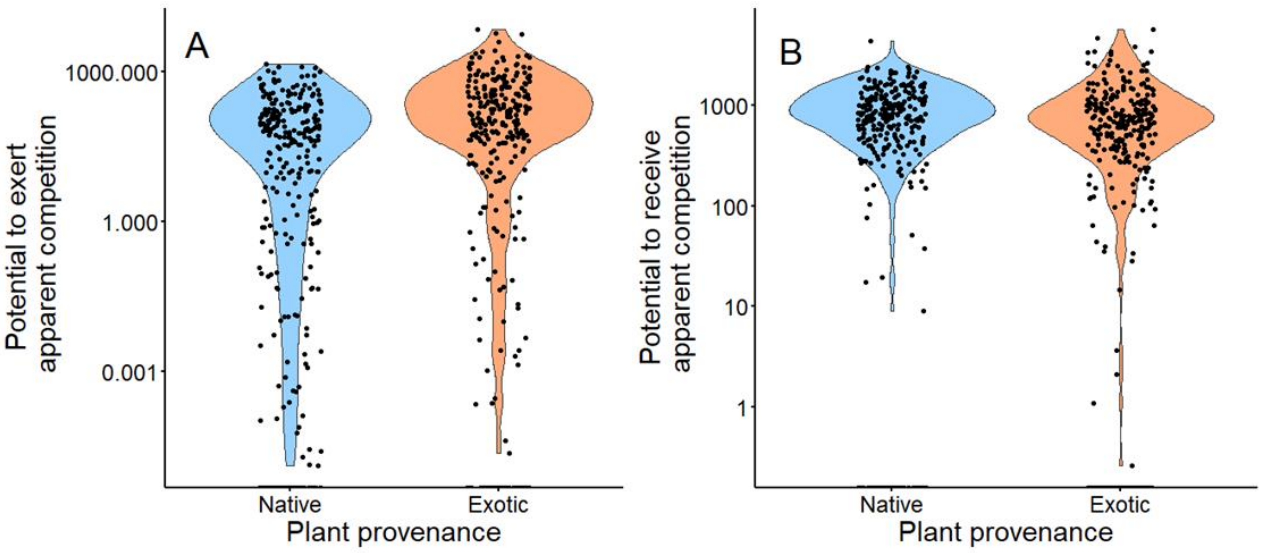

Supplementary Figure 17. Violin plots showing the distribution of raw data underlying the estimated marginal means presented in Fig. 4. Potential for native (blue) and exotic (orange) plants to A) exert and B) receive apparent competition with other plants in the community via shared herbivores. Data in both panels are plotted on a log-scale.

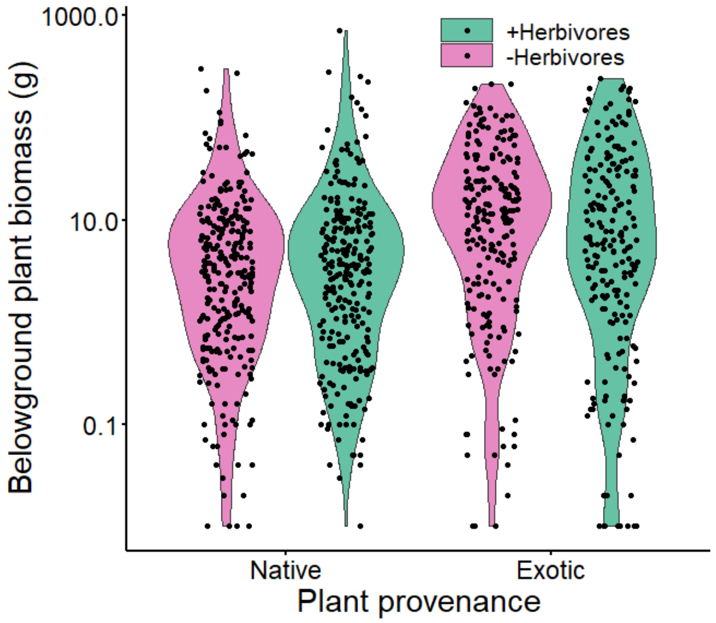

Supplementary Figure 18. Violin plot showing the distribution of raw data underlying the estimated marginal means presented in Supplementary Fig. 1. Belowground biomass of native and exotic plants in mesocosms subjected to different herbivore treatments (pink = -Herbivores; green = +Herbivores). Data is plotted on a log-scale.

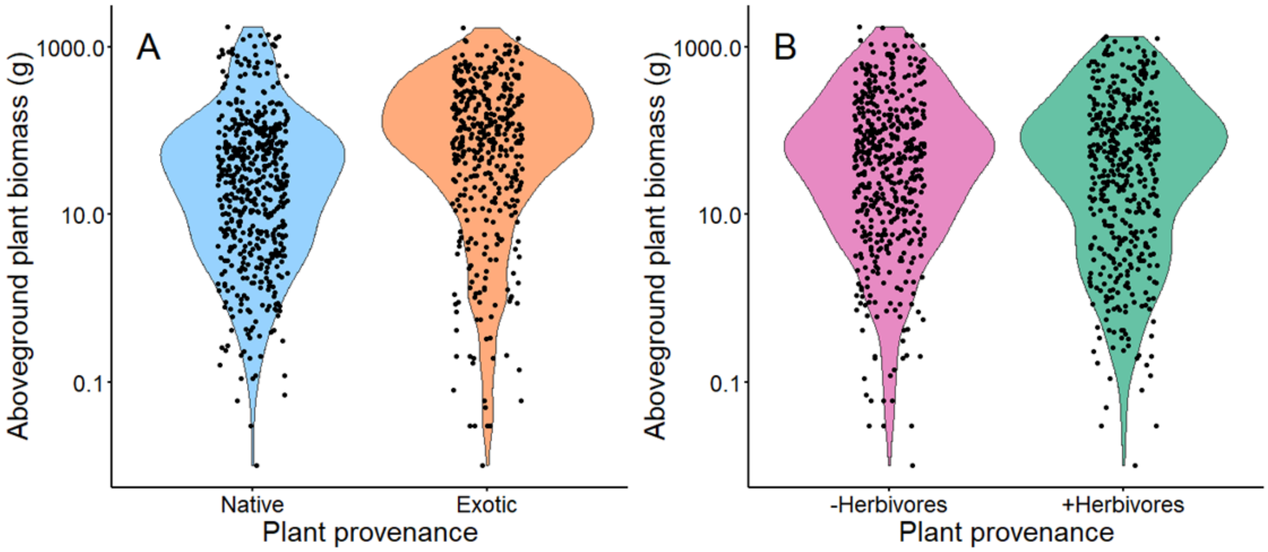

Supplementary Figure 19. Violin plots showing the distribution of raw data underlying the estimated marginal means presented in Supplementary Fig. 2. A) Aboveground biomass of native (blue) and exotic (orange) plants and B) plants in mesocosms subjected to different herbivore treatments (pink = -Herbivores; green = +Herbivores). Data in both panels are plotted on a log-scale.

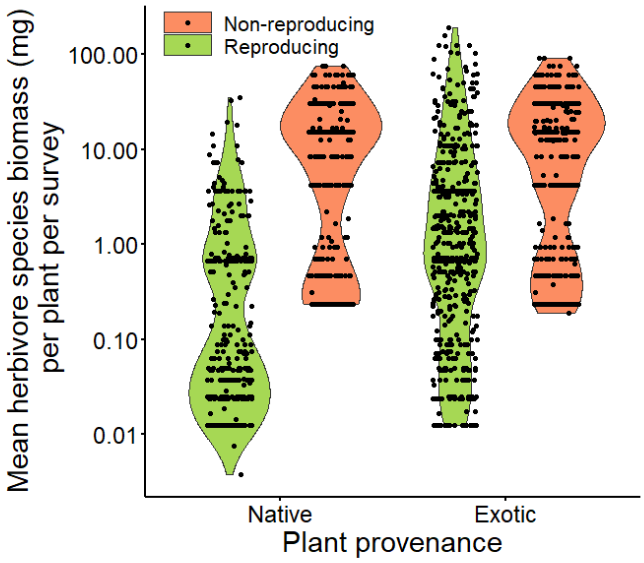

Supplementary Figure 20. Violin plot showing the distribution of raw data underlying the estimated marginal means presented in Supplementary Fig. 7. Herbivore species biomass on native and exotic plant species within their fundamental host range, depending on herbivore reproductive status in the mesocosms (green = non-reproducing; orange = reproducing herbivores). Data is plotted on a log-scale.

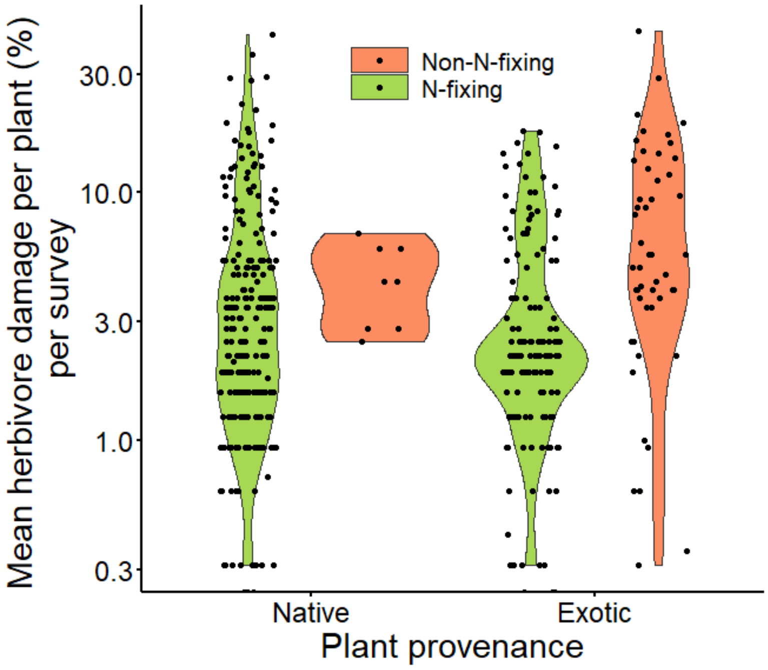

Supplementary Figure 21. Violin plot showing the distribution of raw data underlying the estimated marginal means presented in Supplementary Fig. 10. Herbivore chewing and scraping damage on native and exotic plant species, depending on plant nitrogen-fixing status (green = nitrogen-fixing; orange = non-nitrogen-fixing). Data is plotted on a log-scale.

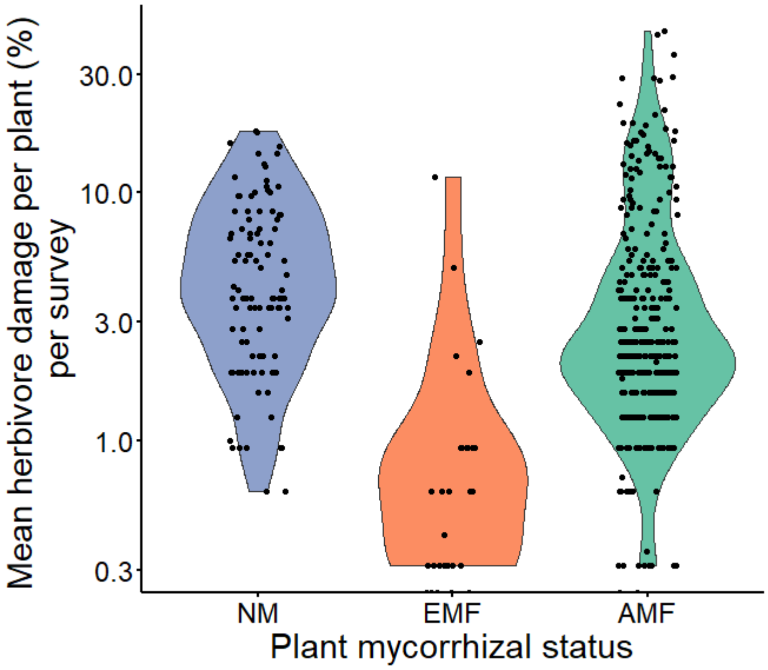

Supplementary Figure 22. Violin plot showing the distribution of raw data underlying the estimated marginal means presented in Supplementary Fig. 11. Herbivore chewing and scraping damage to plant leaf tissue, depending on plant mycorrhizal status (NM, blue = non-mycorrhizal; EMF, orange = ectomycorrhizal fungi associations; AMF, green = arbuscular mycorrhizal fungi associations. Data is plotted on a log-scale.

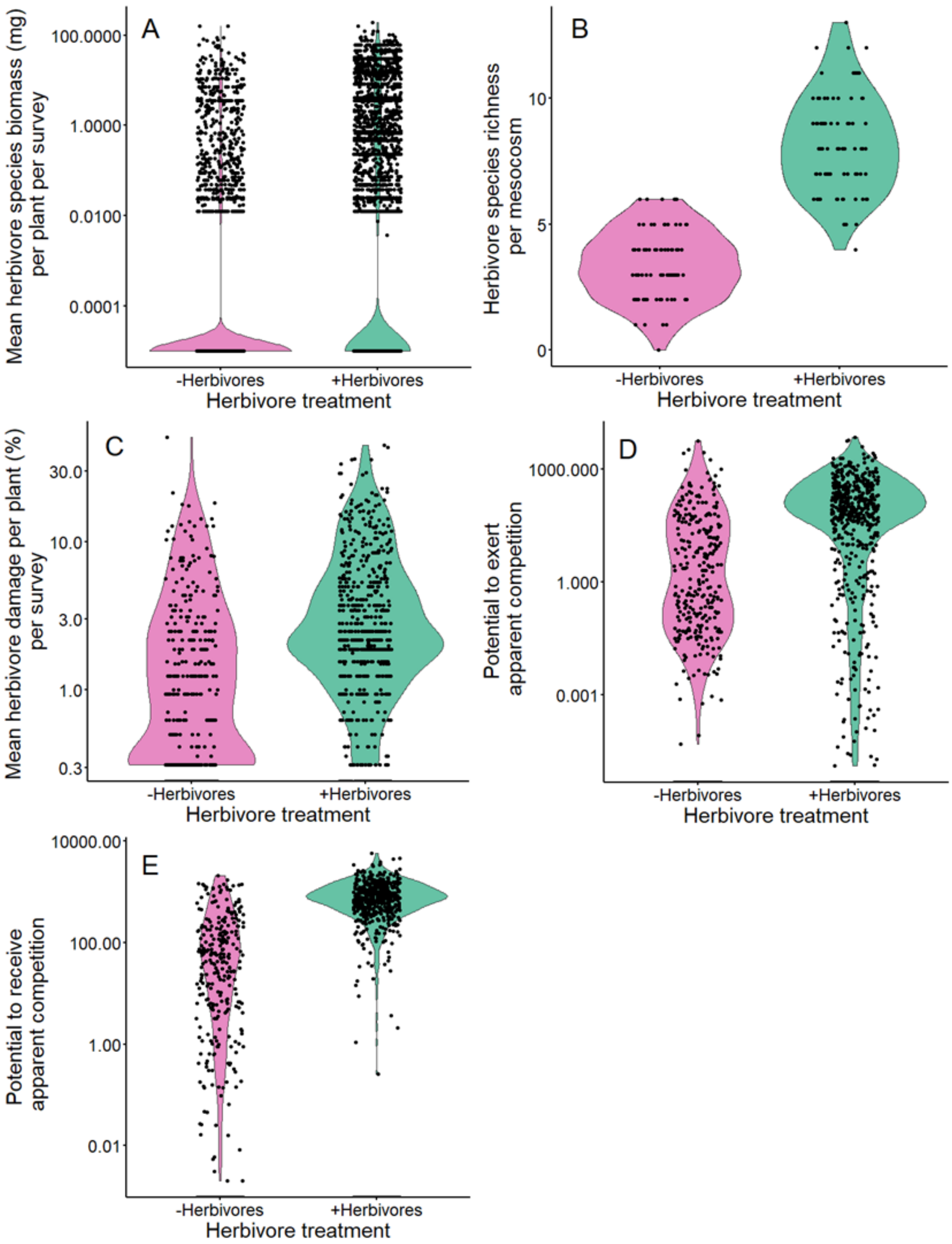

Supplementary Figure 23. Violin plots showing the distribution of raw data underlying the estimated marginal means presented in Supplementary Fig. 14. A) Herbivore species biomass; B) richness; C) chewing and scraping damage; and the potential for plants to D) exert and E) receive apparent competition with other plants via shared herbivores in

mesocosms subjected to different herbivore treatments (pink = -Herbivores; green = +Herbivores). Data in panels A, C, D, and E are plotted on a log-scale.

Supplementary Tables

Supplementary Table 1. Studies cited in the Meijer et al. (2016)[1] meta-analysis that test the enemy release hypothesis by comparing plant-herbivore interactions between native and exotic plants. Data shown include sample size of host and enemy species, enemy type (guild and degree of specialization), and the different measurements used to assess the enemy release hypothesis (diversity = species richness or diversity of herbivores; abundance = abundance, density, biomass, herbivore development; damage = % damage to plant tissues; impact = quantification of impacts on plant fitness via exclusion experiment; indirect = assessment of indirect interactions such as apparent competition).

				Measurement type(s)				
Reference	No. hosts (native/exotic)	No. enemies (native/exotic)	Enemy types	Diversity	Abundance	Damage	Impact	Indirect
This study	19/20	7/13	Mostly polyphagous herbivores from multiple guilds (chewers, suckers, scrapers, leaf-miners)	✓	✓	✓	✓	✓

Agrawal & Kotanen (2003)[2]	15/15	0/1	Whole community (chewers); polyphagous chewer *Spodoptera exigua* (bioassay)	X	X	✓	X	X
Agrawal et al. (2005)[3]	14/14	NA	Whole community	✓	✓	✓	X	X
Agrawal et al. (2005)[3]	1/1	NA	Whole community	X	X	✓	✓	X
Auerbach & Simberloff (1988)[4]	1/2	NA	Leaf-miner community	✓	✓	X	X	X
Bürki & Nentwig (1997)[5]	1/1	NA	Whole community	✓	X	X	X	X
Carpenter & Cappuccino (2005)[6]	30/39	NA	Whole community (chewers)	X	X	✓	X	X
Cincotta et al. (2008)[7]	1/1	NA	Whole community of gallers, chewers, skeletonisers,	✓	✓	✓	X	X

			suckers, and leaf-miners					
Engelkes et al. (2012)[8]	2/2	NA	Whole community of gallers, chewers, suckers, and leaf-miners	X	✓	X	X	X
Goßner et al. (2007)[9]	4/2	NA	Sawfly community	✓	✓	X	X	X
Hartley et al. (2010)[10]	3/1	NA	Whole community; chewer and leaf-miner damage	✓	✓	✓	X	X
Harvey et al. (2015)[11]	5/5	NA	Leaf-miner, sucker, chewer and galler community	✓	✓	✓	X	X
Heard & Sax (2013)[12]	6/6	NA	Whole community	X	X	✓	✓	X
Helden et al. (2012)[13]	16/7	NA	Hemiptera community	✓	✓	X	X	X

Hill & Kotanen (2010)[14]	20/15	NA	Whole community of chewers and leaf-miners	X	X	✓	X	X
Jobin et al. (1996)[15]	1/1	NA	Whole community of gallers, chewers, suckers, and leaf-miners	✓	✓	X	X	X
Kennedy & Southwood (1984)[16]	21/7	NA	Whole community of gallers, chewers, suckers, and leaf-miners	✓	X	X	X	X
Leather (1986)[17]	46/13	NA	Whole community of gallers, chewers, and suckers	✓	X	X	X	X
Lieurance & Cipollini (2013)[18]	1/1	1/1	Whole community (survey);	X	✓	✓	X	X

			oligophagous sawfly *Zaraea inflata* and polyphagous chewer *Spodoptera frugiperda* (bioassays)					
Liu & Stiling (2006)[19]	2/1	NA	Whole community	X	X	✓	X	X
Liu et al. (2007)[20]	2/4	NA	Whole community	X	X	✓	X	X
Lombardero et al. (2008)[21]	1/1	1/0	Pine beetle (*Tomicus piniperda*)	X	✓	✓	X	X
Meijer et al. (2015)[22]	6/11	NA	Whole community	X	X	✓	X	X
Meijer et al. (2015)[22]	12/4	NA	Whole community	X	X	✓	X	X
Meijer et al. (2015)[22]	8/20	NA	Whole community	✓	✓	X	X	X
Meijer et al. (2015)[22]	19/19	NA	Whole community	✓	✓	X	X	X

Novotny et al. (2003)[23]	1/2	NA	Lepidoptera caterpillar community	✓	✓	X	X	X
Procheş et al. (2008)[24]	3/9	NA	Whole community	✓	✓	X	X	X
Radho-Toly et al. (2001)[25]	2/2	NA	Whole community of gallers, chewers, scrapers, and leaf-miners	X	✓	✓	X	X
Schutzenhofer et al. (2009)[26]	1/1	NA	Whole community	X	X	✓	✓	X
Southwood et al. (1982)[27]	4/2	NA	Whole community	✓	✓	X	X	X
Southwood et al. (1982)[27]	3/3	NA	Whole community	✓	✓	X	X	X
Southwood et al. (2004)[28]	2/2	NA	Whole community of gallers, chewers, suckers, and leaf-miners	✓	✓	X	X	X

Sugiura (2010)[29]	102/49	NA	Galler and leaf-miner community	X	✓	X	X	X
Yela & Lawton (1997)[30]	8/3	NA	Lepidoptera and Hymenoptera caterpillar community	X	✓	X	X	X
Zuefle et al. (2008)[31]	15/30	NA	Whole community	✓	✓	X	X	X

Supplementary Table 2. Analysis of variance table for a generalized linear mixed model that aimed to explain variation in the presence of herbivore species per plant. Explanatory variables: P = plant provenance, I = insect provenance, S = soil treatment. Bold text denotes statistically significant variables ($P < 0.05$).

Explanatory variable	Sum of squares	Mean squares	*F*	*P*
P	5.93	5.93	5.93	**0.015**
I	0.09	0.09	0.09	0.765
S	0.46	0.46	0.46	0.512
P×I	2.57	2.57	2.57	0.110
P×S	0.13	0.13	0.13	0.746
I×S	0.37	0.37	0.37	0.544
P×I×S	1.31	1.31	1.31	0.252

Supplementary Table 3. Analysis of variance table for a linear mixed model that aimed to explain variation in mean invertebrate herbivore species biomass per plant. Explanatory variables: P = plant provenance, I = insect provenance, S = soil treatment. Bold text denotes statistically significant variables ($P < 0.05$).

Explanatory variable	Sum of squares	Mean squares	Numerator d.f.	Denominator d.f.	*F*	*P*
P	20.88	20.88	1	41.0	24.71	**0.00001**
I	0.26	0.26	1	18.9	0.31	0.587
S	0.58	0.58	1	1551.1	0.68	0.410
P×I	0.28	0.28	1	1576.1	0.33	0.568
P×S	2.41	2.41	1	1550.2	2.85	0.091
I×S	0.41	0.41	1	1540.0	0.49	0.486
P×I×S	1.12	1.12	1	1542.4	1.33	0.250

Supplementary Table 4. Analysis of variance table for a linear mixed model that aimed to explain variation in total invertebrate herbivore biomass per mesocosm. Explanatory variables: E = proportion of exotic plants planted in the community, I = insect provenance, S = soil treatment. Bold text denotes statistically significant variables ($P < 0.05$).

Explanatory variable	Sum of squares	Mean squares	Numerator d.f.	Denominator d.f.	*F*	*P*
E	4.89	4.89	1	18	10.72	**0.004**
I	73.91	73.91	1	134	162.04	**$<2.2e^{-16}$**
S	0.00	0.00	1	134	0.001	0.979
E×I	19.92	19.92	1	134	43.67	**$8.4e^{-10}$**
E×S	0.01	0.01	1	134	0.03	0.868
I×S	0.51	0.51	1	134	1.12	0.292
E×I×S	1.23	1.23	1	134	2.69	0.103

Supplementary Table 5. Analysis of variance table for a linear mixed model that aimed to explain variation in the ratio of invertebrate herbivore biomass to plant biomass per plant. Explanatory variables: P = plant provenance, I = insect provenance, S = soil treatment.

Explanatory variable	Sum of squares	Mean squares	Numerator d.f.	Denominator d.f.	*F*	*P*
P	0.79	0.79	1	37.8	1.35	0.253
I	1.64	1.64	1	19.0	2.79	0.111
S	0.18	0.18	1	63.3	0.31	0.580
P×I	1.39	1.39	1	9666.8	2.37	0.124
P×S	0.24	0.24	1	244.1	0.41	0.524
I×S	0.05	0.05	1	9666.8	0.08	0.771
P×I×S	1.34	1.34	1	9666.8	2.28	0.131

Supplementary Table 6. Analysis of variance table for a linear mixed model that aimed to explain variation in the ratio of herbivore biomass to plant biomass per mesocosm. Explanatory variables: E = proportion of exotic plants planted in the community, I = insect provenance, S = soil treatment. Bold text denotes statistically significant variables (P < 0.05).

Explanatory variable	Sum of squares	Mean squares	Numerator d.f.	Denominator d.f.	*F*	*P*
E	6.08	6.08	1	18.00	11.55	**0.003**
I	73.92	73.92	1	75.98	140.46	**<2.2e^{-16}**
S	0.02	0.02	1	58.00	0.04	0.852
E×I	19.93	19.93	1	75.98	37.86	**3.3e^{-8}**
E×S	0.40	0.40	1	58.00	076	0.387
I×S	0.51	0.51	1	75.98	0.97	0.328
E×I×S	1.23	1.23	1	75.98	2.33	0.131

Supplementary Table 7. Analysis of variance table for a linear mixed model that aimed to explain variation in normalized degree per plant (i.e., the proportion of species interactions observed out of all possible interactions). Explanatory variables: P = plant provenance, S = soil treatment.

Explanatory variable	Sum of squares	Mean squares	Numerator d.f.	Denominator d.f.	*F*	*P*
P	0.05	0.05	1	48.48	1.35	0.251
S	0.01	0.01	1	108.32	0.22	0.637
P×S	0.01	0.01	1	393.43	0.34	0.559

Supplementary Table 8. Analysis of variance table for a generalized linear mixed model that aimed to explain variation in herbivore species richness per mesocosm. Explanatory variables: E = proportion of exotic plants planted in the community, S = soil treatment. Bold text denotes statistically significant variables ($P < 0.05$).

Explanatory variable	Sum of squares	Mean squares	*F*	*P*
E	9.65	9.65	9.65	**0.002**
S	0.002	0.002	0.002	0.907
E×S	0.35	0.35	0.35	0.552

Supplementary Table 9. Analysis of variance table for a generalized linear mixed model that aimed to explain variation in herbivore chewing and scraping damage per plant. Explanatory variables: P = plant provenance, S = soil treatment.

Explanatory variable	Sum of squares	Mean squares	*F*	*P*
P	0.53	0.53	12.76	0.062
S	0.001	0.001	0.03	0.891
P×S	0.0003	0.0003	0.01	0.930

Supplementary Table 10. Analysis of variance table for a generalized linear mixed model that aimed to explain variation in the mean herbivore chewing and scraping damage per plant for each mesocosm. Explanatory variables: E = proportion of exotic plants planted in the community, S = soil treatment.

Explanatory variable	Sum of squares	Mean squares	*F*	*P*
E	0.53	0.53	6.53	0.116
S	0.07	0.07	0.83	0.393
E×S	0.01	0.01	0.13	0.733

Supplementary Table 11. Analysis of variance table for a linear mixed model that aimed to explain variation in individual total plant biomass. Explanatory variables: P = plant provenance, S = soil treatment, H = herbivore treatment. Bold text denotes statistically significant variables ($P < 0.05$).

Explanatory variable	Sum of squares	Mean square	Numerator d.f.	Denominator d.f.	*F*	*P*
P	8.27	8.27	1	36.29	5.61	**0.023**
S	1.46	1.46	1	885.09	0.99	0.320
H	10.49	10.49	1	884.25	7.12	**0.008**
P×S	0.56	0.56	1	885.17	0.38	0.538
P×H	6.00	6.00	1	884.21	4.08	**0.044**
S×H	0.01	0.01	1	884.17	0.01	0.923
P×S×H	0.06	0.06	1	883.97	0.04	0.839

Supplementary Table 12. Analysis of variance table for a linear mixed model that aimed to explain variation in individual belowground plant biomass. Explanatory variables: P = plant provenance, S = soil treatment, H = herbivore treatment. Bold text denotes statistically significant variables ($P < 0.05$).

Explanatory variable	Sum of squares	Mean square	Numerator d.f.	Denominator d.f.	*F*	*P*
P	12.45	12.45	1	35.31	6.83	**0.013**
S	0.34	0.34	1	884.15	0.19	0.665
H	13.19	13.19	1	882.99	7.24	**0.007**
P×S	0.44	0.44	1	884.28	0.24	0.624
P×H	15.03	15.03	1	882.93	8.25	**0.004**
S×H	1.78	1.78	1	882.82	0.97	0.323
P×S×H	0.004	0.004	1	882.60	0.002	0.964

Supplementary Table 13. Analysis of variance table for a linear mixed model that aimed to explain variation in individual aboveground plant biomass. Explanatory variables: P = plant provenance, S = soil treatment, H = herbivore treatment. Bold text denotes statistically significant variables ($P < 0.05$).

Explanatory variable	Sum of squares	Mean square	Numerator d.f.	Denominator d.f.	*F*	*P*
P	8.84	8.84	1	36.36	5.52	**0.024**
S	2.28	2.28	1	885.00	1.43	0.233
H	12.10	12.10	1	884.12	7.56	**0.006**
P×S	1.10	1.10	1	885.08	0.69	0.408
P×H	5.67	5.67	1	884.07	3.54	0.060
S×H	0.01	0.01	1	884.03	0.01	0.939
P×S×H	0.01	0.01	1	883.83	0.005	0.944

Supplementary Table 14. Analysis of variance table for a linear mixed model that aimed to explain variation in total plant biomass per mesocosm. Explanatory variables: H = herbivore treatment, S = soil treatment, E = proportion of exotic plants planted in the community. Bold text denotes statistically significant variables ($P < 0.05$).

Explanatory variable	Sum of squares	Mean square	Numerator d.f.	Denominator d.f.	*F*	*P*
H	0.03	0.03	1	134	0.29	0.588
S	0.002	0.002	1	134	0.03	0.868
E	0.17	0.17	1	18	1.69	0.210
H×S	0.01	0.01	1	134	0.06	0.804
H×E	0.0004	0.0004	1	134	0.004	0.953
S×E	0.44	0.44	1	134	4.27	**0.041**
H×S×E	0.01	0.01	1	134	0.06	0.813

Supplementary Table 15. Analysis of variance table for a linear mixed model that aimed to explain variation in belowground plant biomass per mesocosm. Explanatory variables: H = herbivore treatment, S = soil treatment, E = proportion of exotic plants planted in the community. Bold text denotes statistically significant variables ($P < 0.05$).

Explanatory variable	Sum of squares	Mean square	Numerator d.f.	Denominator d.f.	*F*	*P*
H	0.40	0.40	1	134	1.22	0.272
S	0.07	0.07	1	134	0.20	0.656
E	1.03	1.03	1	18	3.10	0.096
H×S	0.001	0.001	1	134	0.004	0.952
H×E	0.46	0.46	1	134	1.40	0.239
S×E	1.64	1.64	1	134	4.93	**0.028**
H×S×E	0.001	0.001	1	134	0.003	0.956

Supplementary Table 16. Analysis of variance table for a linear mixed model that aimed to explain variation in aboveground plant biomass per mesocosm. Explanatory variables: H = herbivore treatment, S = soil treatment, E = proportion of exotic plants planted in the community.

Explanatory variable	Sum of squares	Mean square	Numerator d.f.	Denominator d.f.	*F*	*P*
H	0.09	0.09	1	134	0.81	0.369
S	0.001	0.001	1	134	0.01	0.942
E	0.10	0.10	1	18	0.94	0.345
H×S	0.002	0.002	1	134	0.01	0.903
H×E	0.002	0.002	1	134	0.01	0.903
S×E	0.41	0.41	1	134	3.73	0.056
H×S×E	0.01	0.01	1	134	0.05	0.823

Supplementary Table 17. Analysis of variance table for a linear mixed model that aimed to explain variation in the potential for each plant to exert apparent competition ($PAC_{exerted}$) on the rest of the community via shared herbivores. Explanatory variables: P = plant provenance, S = soil treatment. Bold text denotes statistically significant variables ($P < 0.05$).

Explanatory variable	Sum of squares	Mean squares	Numerator d.f.	Denominator d.f.	*F*	*P*
P	75.90	75.90	1	40.39	7.07	**0.011**
S	19.38	19.38	1	34.96	1.81	0.188
P×S	12.60	12.60	1	142.86	1.17	0.280

Supplementary Table 18. Analysis of variance table for a linear mixed model that aimed to explain variation in the potential for each plant to receive apparent competition ($PAC_{received}$) from the rest of the community via shared herbivores. Explanatory variables: P = plant provenance, S = soil treatment.

Explanatory variable	Sum of squares	Mean squares	Numerator d.f.	Denominator d.f.	*F*	*P*
P	4.30	4.30	1	38.40	0.32	0.575
S	3.91	3.91	1	58.76	0.29	0.591
P×S	8.30	8.30	1	299.64	0.62	0.432

Supplementary Table 19. Analysis of variance table for a generalized linear mixed model that aimed to explain variation in the presence of herbivore species per plant using plant and herbivore traits. Explanatory variables: P = plant provenance, B = plant total biomass, N = plant nitrogen-fixing status, M = plant mycorrhizal status, F = plant functional group, S = plant specific leaf area, R = insect reproduction status. Bold text denotes statistically significant variables ($P < 0.05$).

Explanatory variable	Sum of squares	Mean square	d.f.	*F*	*P*
P	35.75	35.75	1	35.75	**0.0003**
F	45.23	15.08	3	15.07	0.296
M	3.00	1.50	2	1.50	0.297
N	15.29	15.29	1	15.29	**0.002**
S	0.80	0.80	1	0.80	0.331
B	24.95	24.95	1	24.95	**0.00003**
R	0.10	0.10	1	0.10	0.755
P×F	1.20	0.40	3	0.40	0.861
P×M	6.02	3.01	2	3.01	0.080
P×N	0.31	0.31	1	0.31	0.522
P×S	0.05	0.05	1	0.05	0.989

P×B	1.39	1.39	1	1.39	0.278
P×R	6.00	6.00	1	6.00	**0.015**

Supplementary Table 20. Analysis of variance table for a linear mixed model that aimed to explain variation in the mean invertebrate herbivore species biomass per plant using plant and herbivore traits. Explanatory variables: P = plant provenance, B = plant total biomass, N = plant nitrogen-fixing status, M = plant mycorrhizal status, F = plant functional group, S = plant specific leaf area, R = insect reproduction status. Bold text denotes statistically significant variables ($P < 0.05$).

Explanatory variable	Sum of squares	Mean square	Numerator d.f.	Denominator d.f.	*F*	*P*
P	0.29	0.29	1	53.42	0.3	0.560
F	2.54	0.85	3	32.36	0.99	0.408
M	5.49	2.75	2	24.47	3.22	0.057
N	0.03	0.03	1	72.10	0.03	0.856
S	2.01	2.01	1	19.43	2.36	0.140
B	9.68	9.68	1	399.71	11.35	**0.001**
R	4.16	4.16	1	18.36	4.88	**0.040**
P×F	3.87	1.29	3	32.46	1.51	0.230
P×M	2.03	1.02	2	24.53	1.19	0.320
P×N	0.45	0.45	1	71.31	0.52	0.471
P×S	0.06	0.06	1	19.23	0.07	0.797

P×B	3.49	3.49	1	381.10	4.10	**0.044**
P×R	56.90	56.90	1	1405.62	66.76	**$6.8e^{-16}$**

Supplementary Table 21. Analysis of variance table for a linear mixed model that aimed to explain variation in normalized degree per plant (i.e., the proportion of species interactions observed out of all possible interactions) using plant and herbivore traits. Explanatory variables: P = plant provenance, B = plant total biomass, N = plant nitrogen-fixing status, M = plant mycorrhizal status, F = plant functional group, S = plant specific leaf area. Bold text denotes statistically significant variables (*P* < 0.05).

Explanatory variable	Sum of squares	Mean square	Numerator d.f.	Denominator d.f.	*F*	*P*
P	0.0001	0.0001	1	26.43	0.01	0.940
F	0.19	0.06	3	21.30	2.65	0.075
M	0.03	0.01	2	19.83	0.54	0.589
N	0.02	0.02	1	30.51	0.80	0.378
S	0.01	0.01	1	18.49	0.33	0.572
B	0.52	0.52	1	349.51	22.35	**0.00003**
P×F	0.02	0.01	3	21.30	0.27	0.847
P×M	0.01	0.004	2	20.36	0.15	0.860
P×N	0.001	0.001	1	30.60	0.04	0.835
P×S	0.01	0.01	1	18.05	0.52	0.481
P×B	0.12	0.12	1	348.01	5.01	**0.026**

Supplementary Table 22. Analysis of variance table for a generalized linear mixed model that aimed to explain variation in herbivore chewing and scraping damage per plant using plant and herbivore traits. Explanatory variables: P = plant provenance, B = plant total biomass, N = plant nitrogen-fixing status, M = plant mycorrhizal status, F = plant functional group, S = plant specific leaf area. Bold text denotes statistically significant variables ($P < 0.05$).

Explanatory variable	Sum of squares	Mean square	d.f.	*F*	*P*
P	0.10	0.10	1	3.39	0.785
F	0.28	0.09	3	3.23	0.304
M	1.49	0.74	2	25.90	**0.003**
N	0.19	0.19	1	6.73	**0.029**
S	0.04	0.04	1	1.52	0.402
B	0.01	0.01	1	0.31	0.641
P×F	0.38	0.13	3	4.38	0.211
P×M	0.07	0.04	2	1.24	0.299
P×N	0.26	0.26	1	8.96	**0.043**
P×S	0.28	0.28	1	9.69	0.090

P×B	0.02	0.02	1	0.71	0.376

Supplementary Table 23. Plant species composition of each mesocosm community.

Community	Plant species	Community	Plant species	Community	Plant species
1	*Agrostis capillaris*	2	*Achillea millefolium*	3	*Acaena inermis*
1	*Anthoxanthum odoratum*	2	*Cirsium vulgare*	3	*Anthoxanthum odoratum*
1	*Holcus lanatus*	2	*Dactylis glomerata*	3	*Brachyglottis greyi*
1	*Hypericum perforatum*	2	*Echium vulgare*	3	*Chionochloa conspicua*
1	*Lolium perenne*	2	*Festuca novae-zelandiae*	3	*Echium vulgare*
1	*Rumex obtusifolius*	2	*Medicago sativa*	3	*Poa colensoi*
1	*Trifolium pratense*	2	*Ozothamnus leptophyllus*	3	*Rumex acetosella*
1	*Trifolium repens*	2	*Rumex acetosella*	3	*Rumex obtusifolius*
4	*Acaena caesiiglauca*	5	*Acaena caesiiglauca*	6	*Achillea millefolium*
4	*Anemanthele lessoniana*	5	*Acaena inermis*	6	*Cirsium vulgare*
4	*Carex secta*	5	*Anemanthele lessoniana*	6	*Holcus lanatus*
4	*Festuca novae-zelandiae*	5	*Brachyglottis greyi*	6	*Lolium perenne*
4	*Hypericum perforatum*	5	*Festuca novae-zelandiae*	6	*Lupinus arboreus*
4	*Medicago sativa*	5	*Ozothamnus leptophyllus*	6	*Pinus contorta*

4	*Phormium cookianum*	5	*Phormium cookianum*	6	*Trifolium pratense*
4	*Poa cita*	5	*Poa colensoi*	6	*Trifolium repens*
7	*Achillea millefolium*	8	*Acacia dealbata*	9	*Anemanthele lessoniana*
7	*Agrostis capillaris*	8	*Acaena caesiiglauca*	9	*Anthoxanthum odoratum*
7	*Carex secta*	8	*Acaena inermis*	9	*Brachyglottis greyi*
7	*Dactylis glomerata*	8	*Alnus glutinosa*	9	*Carex secta*
7	*Hypericum perforatum*	8	*Cirsium vulgare*	9	*Coprosma robusta*
7	*Ozothamnus leptophyllus*	8	*Phormium cookianum*	9	*Festuca novae-zelandiae*
7	*Pinus radiata*	8	*Poa cita*	9	*Muehlenbeckia astonii*
7	*Ulex europaeus*	8	*Trifolium pratense*	9	*Rumex acetosella*
10	*Acaena caesiiglauca*	11	*Acacia dealbata*	12	*Alnus glutinosa*
10	*Carex secta*	11	*Agrostis capillaris*	12	*Anemanthele lessoniana*
10	*Festuca novae-zelandiae*	11	*Dactylis glomerata*	12	*Brachyglottis greyi*
10	*Leptospermum scoparium*	11	*Holcus lanatus*	12	*Echium vulgare*
10	*Olearia virgata*	11	*Lolium perenne*	12	*Lupinus arboreus*
10	*Ozothamnus leptophyllus*	11	*Pinus radiata*	12	*Medicago sativa*

10	*Phormium cookianum*	11	*Trifolium repens*	12	*Pinus contorta*
10	*Poa cita*	11	*Ulex europaeus*	12	*Rumex obtusifolius*
13	*Acacia dealbata*	14	*Acaena inermis*	15	*Acaena inermis*
13	*Achillea millefolium*	14	*Anemanthele lessoniana*	15	*Carex secta*
13	*Carex secta*	14	*Echium vulgare*	15	*Leptospermum scoparium*
13	*Hypericum perforatum*	14	*Holcus lanatus*	15	*Ozothamnus leptophyllus*
13	*Leptospermum scoparium*	14	*Muehlenbeckia complexa*	15	*Phormium cookianum*
13	*Poa colensoi*	14	*Poa cita*	15	*Poa cita*
13	*Rumex obtusifolius*	14	*Podocarpus totara*	15	*Sophora microphylla*
13	*Hebe odora*	14	*Sophora microphylla*	15	*Hebe odora*
16	*Agrostis capillaris*	17	*Anthoxanthum odoratum*	18	*Alnus glutinosa*
16	*Alnus glutinosa*	17	*Coprosma robusta*	18	*Brachyglottis greyi*
16	*Lupinus arboreus*	17	*Lolium perenne*	18	*Cirsium vulgare*
16	*Pinus contorta*	17	*Lupinus arboreus*	18	*Lupinus arboreus*
16	*Pinus radiata*	17	*Ozothamnus leptophyllus*	18	*Muehlenbeckia complexa*
16	*Rumex acetosella*	17	*Pinus contorta*	18	*Olearia virgata*

16	*Trifolium pratense*	17	*Pinus radiata*	18	*Phormium cookianum*
16	*Ulex europaeus*	17	*Ulex europaeus*	18	*Ulex europaeus*
19	*Acaena caesiiglauca*	20	*Carex secta*		
19	*Muehlenbeckia astonii*	20	*Muehlenbeckia astonii*		
19	*Muehlenbeckia complexa*	20	*Olearia virgate*		
19	*Phormium cookianum*	20	*Ozothamnus leptophyllus*		
19	*Pinus contorta*	20	*Phormium cookianum*		
19	*Pinus radiata*	20	*Podocarpus totara*		
19	*Poa colensoi*	20	*Sophora microphylla*		
19	*Podocarpus totara*	20	*Hebe odora*		

Supplementary Table 24. All plant species used in the experiment. Information shown includes plant species' provenance, weed status on conservation (Con.) or agricultural (Agr.) land (No = not a weed according to published lists, - = native plant species)[32,33], functional group, and the number of mesocosms they were planted into.

Plant name	Family	Provenance	Weed status	Functional group	# mesocosms	Fig. 5 symbol
Acacia dealbata	Fabaceae	Exotic	Con.	Woody	24	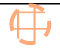
Acaena caesiiglauca	Rosaceae	Native	-	Herbaceous	40	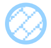
Acaena inermis	Rosaceae	Native	-	Herbaceous	40	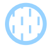
Achillea millefolium	Asteraceae	Exotic	Agr.	Herbaceous	32	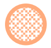
Agrostis capillaris	Poaceae	Exotic	Agr., Con.	Herbaceous	32	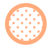
Alnus glutinosa	Betulaceae	Exotic	Con.	Woody	32	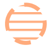
Anemanthele lessoniana	Poaceae	Native	-	Herbaceous	40	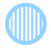
Anthoxanthum odoratum	Poaceae	Exotic	Agr., Con.	Herbaceous	32	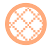
Brachyglottis greyi	Asteraceae	Native	-	Herbaceous	40	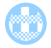
Carex secta	Cyperaceae	Native	-	Herbaceous	56	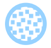
Chionochloa conspicua	Poaceae	Native	-	Herbaceous	8	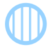
Cirsium vulgare	Asteraceae	Exotic	Agr., Con.	Herbaceous	32	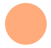

Coprosma robusta	Rubiaceae	Native	-	Woody	16
Dactylis glomerata	Poaceae	Exotic	Con.	Herbaceous	24
Echium vulgare	Boraginaceae	Exotic	Agr., Con.	Herbaceous	32
Festuca novae-zelandiae	Poaceae	Native	-	Herbaceous	40
Hebe odora	Plantaginaceae	Native	-	Woody	24
Holcus lanatus	Poaceae	Exotic	Agr., Con.	Herbaceous	32
Hypericum perforatum	Hypericaceae	Exotic	Agr., Con.	Herbaceous	32
Leptospermum scoparium	Myrtaceae	Native	-	Woody	24
Lolium perenne	Poaceae	Exotic	Con.	Herbaceous	32
Lupinus arboreus	Fabaceae	Exotic	Con.	Woody	40
Medicago sativa	Fabaceae	Exotic	No	Herbaceous	24
Muehlenbeckia astonii	Polygonaceae	Native	-	Woody	24
Muehlenbeckia complexa	Polygonaceae	Native	-	Herbaceous	24
Olearia virgata	Asteraceae	Native	-	Woody	24
Ozothamnus leptophyllus	Asteraceae	Native	-	Herbaceous	56
Phormium cookianum	Asphodelaceae	Native	-	Herbaceous	64

Pinus contorta	Pinaceae	Exotic	Con.	Woody	40	
Pinus radiata	Pinaceae	Exotic	Con.	Woody	40	
Poa cita	Poaceae	Native	-	Herbaceous	40	
Poa colensoi	Poaceae	Native	-	Herbaceous	32	
Podocarpus totara	Podocarpaceae	Native	-	Woody	24	
Rumex acetosella	Polygonaceae	Exotic	Agr.	Herbaceous	32	
Rumex obtusifolius	Polygonaceae	Exotic	Agr.	Herbaceous	32	
Sophora microphylla	Fabaceae	Native	-	Woody	24	
Trifolium pratense	Fabaceae	Exotic	No	Herbaceous	32	
Trifolium repens	Fabaceae	Exotic	Con.	Herbaceous	24	
Ulex europaeus	Fabaceae	Exotic	Agr., Con.	Woody	40	

Supplementary Table 25. All herbivore species used in the mesocosm experiment. Information shown includes herbivore species' provenance (Prov.), feeding guild, degree of specialization, number of mesocosms colonized (# meso), number of host plant species fed on in the mesocosms (# hosts), and whether each species self-colonized, successfully established, and reproduced within mesocosms. Y = Yes, N = No.

Species name	Order: Family	Prov.	Guild	Specialization	# meso.	# hosts	Self-colonizer	Established	Reproduced
Costelytra giveni	Coleoptera: Scarabaeidae	Native	Root/leaf chewer	Polyphagous	55	27	N	Y	N
Naupactus godmanni	Coleoptera: Curculionidae	Exotic	Root/leaf chewer	Polyphagous	2	3	Y	Y	N
Sitona obsoletus	Coleoptera: Curculionidae	Exotic	Root/leaf chewer	Oligophagous	41	2	N	Y	N
Sitona discoideus	Coleoptera: Curculionidae	Exotic	Leaf chewer	Oligophagous	13	1	N	Y	N
Lema cyanella	Coleoptera: Chrysomelidae	Exotic	Leaf chewer	Oligophagous	3	1	Y	Y	N
Epiphyas postvittana	Lepidoptera: Tortricidae	Exotic	Leaf chewer	Polyphagous	85	31	N	Y	Y
Ctenopseustis obliquana	Lepidoptera: Tortricidae	Native	Leaf chewer	Polyphagous	41	23	N	Y	Y
Planotortrix excessana	Lepidoptera: Tortricidae	Native	Leaf chewer	Polyphagous	45	21	N	Y	Y
Dialectica scalariella	Lepidoptera: Gracillariidae	Exotic	Leaf miner	Oligophagous	1	1	N	Y	N
Agrotis ipsilon	Lepidoptera: Noctuidae	Native	Leaf chewer	Polyphagous	5	3	Y	Y	N
Pseudocoremia suavis	Lepidoptera: Geometridae	Native	Leaf chewer	Polyphagous	5	10	Y	Y	N
Anzygina zealandica	Hemiptera: Cicadellidae	Native	Sucker	Polyphagous	145	37	N	Y	Y
Philaenus spumarius	Hemiptera: Aphrophoridae	Exotic	Sucker	Polyphagous	76	30	N	Y	N
Acyrthosiphon pisum	Hemiptera: Aphidide	Exotic	Sucker	Oligophagous	7	3	Y	Y	Y

Rhopalosiphum padi	Hemiptera: Aphidide	Exotic	Sucker	Polyphagous	29	9	N	Y	Y
Myzus persicae	Hemiptera: Aphidide	Exotic	Sucker	Polyphagous	27	16	N	Y	Y
Aulacorthum solani	Hemiptera: Aphidide	Exotic	Sucker	Polyphagous	64	7	N	Y	Y
Paprides nitidus	Orthoptera: Acrididae	Native	Leaf chewer	Polyphagous	80	38	N	Y	N
Sminthurus viridis	Collembola: Sminthuridae	Exotic	Leaf chewer	Polyphagous	92	32	Y	Y	Y
Deroceras sp.	Stylommatophora: Agriolimacidae	Exotic	Leaf chewer	Polyphagous	113	31	Y	Y	Y
Teleogryllus commodus	Orthoptera: Gryllidae	Exotic	Leaf chewer	Polyphagous	0	-	N	N	-
Wiseana copularis	Lepidoptera: Hepialidae	Native	Leaf chewer	Polyphagous	0	-	N	N	-

Supplementary Table 26. Details of statistical models that tested our research questions and specific predictions. Information shown includes response variables and their transformation, error structure, number of observations (n), and explanatory variables used for analyses (main effects are not shown for terms included in an interaction). Explanatory variables: P = plant provenance, I = insect provenance, H = herbivore treatment, S = soil treatment, E = proportion of exotic plants planted in the community, C = plant community, M = mesocosm, Psp = plant species, Isp = insect species, B = plant biomass, $PAC_{received}$ = potential for apparent competition received from the community.

Prediction from table 1	Response variable	Explanatory variables	Transformation	Error structure	n
1a, 4b	Herbivore presence (plant-herbivore level)	Fixed: P×I×S<br>Random: C/M, Psp, Isp	None	Binary	5528
1a, 4b	Herbivore biomass (plant-herbivore level)	Fixed: P×I×S<br>Random: C/M, Psp, Isp	Log	Gaussian	1607
1a, 4b	Herbivore biomass (mesocosm level)	Fixed: E×I×S<br>Random: C/M	Log	Gaussian	160
1a, 4b	Herbivore:plant biomass ratio (plant-herbivore level)	Fixed: P×I×S<br>Random: C/M, Psp, Isp	Log	Gaussian	467
1a, 4b	Herbivore:plant biomass ratio (mesocosm level)	Fixed: E×I×S	Log	Gaussian	160

		Random: C/M			
1b, 4b	Normalized degree (plant level)	Fixed: P×S	None	Gaussian	435
		Random: C/M, Psp			
1b, 4b	Herbivore species richness (mesocosm level)	Fixed: E×S	None	Poisson	80
		Random: C			
1c, 4b	Chewing and scraping damage (plant level)	Fixed: P×S	Logit	Gamma (log link)	640
		Random: C/M, Psp			
1c, 4b	Chewing and scraping damage (mesocosm level)	Fixed: E×S	Logit	Gamma (log link)	80
		Random: C			
2a, 4b	$PAC_{exerted}$ (plant level)	Fixed: P×S	Log	Gaussian	640
		Random: C/M, Psp			
2a, 4b	$PAC_{received}$ (plant level)	Fixed: P×S	Log	Gaussian	640
		Random: C/M, Psp			
2b	Total plant biomass (plant level; impact of PAC)	Fixed: $PAC_{received}$×H	Log	Gaussian	951
		Random: C/M, Psp			
2b	Chewing and scraping damage (plant level; impact of PAC)	Fixed: $PAC_{received}$	Logit	Gamma (log link)	467

		Random: C/M, Psp			
2c	$PAC_{exerted}$ (plant level; plant biomass)	Fixed: B	Log	Gaussian	640
		Random: C/M, Psp			
3a, 4a, 4b	Total plant biomass (plant level)	Fixed: P×S×H	Log	Gaussian	951
		Random: C/M, Psp			
3a, 4a, 4b	Aboveground plant biomass (plant level)	Fixed: P×S×H	Log	Gaussian	951
		Random: C/M, Psp			
3a, 4a, 4b	Belowground plant biomass (plant level)	Fixed: P×S×H	Log	Gaussian	951
		Random: C/M, Psp			
3a, 4a, 4b	Total plant biomass (mesocosm level)	Fixed: E×H×S	Log	Gaussian	160
		Random: C			
3a, 4a, 4b	Aboveground plant biomass (mesocosm level)	Fixed: E×H×S	Log	Gaussian	160
		Random: C			
3a, 4a, 4b	Belowground plant biomass (mesocosm level)	Fixed: E×H×S	Log	Gaussian	160
		Random: C			

Supplementary Methods

Herbivore cage design

Herbivore cages (Supplementary Figure 13) were constructed using Cropsafe Protection Mesh (0.58 mm, 15% shade factor) from Cosio Industries (Auckland, New Zealand), designed to keep out small insects like aphids and psyllids. The mesh was cut and sewn into tubes (255 cm long, 81 cm diameter) with Dabond 25/V92 UV-resistant thread from Coats Industrial (Auckland, New Zealand). The tube shape was reinforced by threading No. 8 wire (4 mm diameter) through loops sewn 75 cm from the top and bottom of the mesh. One open end of each tube was tightly drawn together and closed with cable ties, then hung from an overhead wire. The open bottom of each cage was secured around the mesocosm pot with two bungee cords that were later replaced by 10 cm wide strips of steel closed with a bolt. For access to the mesocosm community, we cut a 50 cm vertical slit in one side of the cage that was closed by tightly folding the mesh over on itself and secured with three 50 mm foldback binder clips.

Herbivore collection, establishment and assessment of feeding interactions

<u>Grass grub, *Costelytra giveni* Coca-Abia & Romero-Samper:</u>

Collections of emerging adult grass grubs were made at dusk on 10 and 23 November 2017 from a garden near Southbridge, New Zealand (43°48' S, 172°15' E). Sex of the adult grass grubs was determined following Kelsey (1965)[34] and Kain (1972)[35], and three females and a single male (due to a natural 3:1 biased sex ratio) were added to the center of each +Herbivore mesocosm the day after each sampling occasion. Females were assumed to have mated at the time of introduction because of the frequent mating observed in the collection containers. Because grass grub adults are short-lived and larvae feed belowground, sampling

could only be conducted during the final mesocosm harvest by thoroughly searching through plant roots and the mesocosm soil as it was homogenized. Because direct observation of larval feeding was impossible, this species was subjected to molecular analysis of gut contents to determine the host plant(s) of each individual (see below for description of these methods). Grass grub larvae were found in 55 of the 80 mesocosms they were introduced to, along with one -Herbivore mesocosm. Based on the Plant-SyNZ Database (a comprehensive database of published plant-insect interactions in New Zealand: https://plant-synz.landcareresearch.co.nz), grass grubs have been recorded feeding on 50 host plant species in New Zealand, and were recorded on 27 plant species in the mesocosms.

Fuller's rose weevil, *Naupactus godmanni* (Crotch):

Fuller's rose weevil was a self-colonizer of two mesocosms and was only detected as larvae at harvest by thoroughly searching through plant roots and the mesocosm soil as it was homogenized. Because Fuller's rose weevil larvae feed belowground and direct observation of larval feeding was impossible, this species was subjected to molecular analysis of gut contents to determine their host plant (see below). Based on the Plant-SyNZ Database, Fuller's rose weevil have been recorded feeding on 17 host plant species in New Zealand, and were recorded on 3 plant species in the mesocosms.

Clover root weevil, *Sitona obsoletus* (Gmelin) and Lucerne weevil, *Sitona discoideus* Gyllenhal:

On 30-31 May 2017, clover root weevils and lucerne weevils were collected using a vacuum sampler from patches of clover (*Trifolium* sp.) and a field of lucerne (*Medicago sativa*), respectively, that were adjacent to the experimental site. Because a significant proportion of adult weevils are likely to be parasitized by a biological control agent

(*Microctonus aethiopoides*, Hymenoptera: Braconidae), they were maintained for three weeks to purge parasitized individuals, before four weevils (two of each sex, determined by examining the shape of the posterior ventrite following Bright (1994)[36]) were added to the center of each mesocosm. Because direct feeding of adults was difficult to observe, their presence was determined by characteristic match-head size notches on clover leaf margins. Moreover, because clover root weevil and lucerne weevil larvae feed belowground, sampling could only be conducted during the final mesocosm harvest by thoroughly searching through plant roots and the mesocosm soil as it was homogenized. Clover root weevil larvae were encountered in only a single mesocosm (host confirmed as red clover, *Trifolium pratense*, using RFLP analysis) and no lucerne weevil larvae were found. These two weevils are oligophagous, with narrow host plant ranges. Clover root weevil has been recorded feeding on 6 *Trifolium* species in New Zealand and lucerne weevil on 4 *Medicago* species (based on data from the Plant-SyNZ Database), although a global review showed that their fundamental host plant ranges are broader[37].

Thistle leaf beetle, *Lema cyanella* (L.):

Thistle leaf beetle is an exotic biological control agent that self-colonized three mesocosms, where it fed exclusively on Scotch thistle (*Cirsium vulgare*) and was found in low abundance as larvae only. This species was removed from -Herbivore mesocosms when encountered and left alone when found in +Herbivore mesocosms. Based on the Plant-SyNZ Database, thistle leaf beetle has been found feeding on a single thistle species in New Zealand (*Cirsium arvense*), but we recorded small numbers of larvae on four *Cirsium vulgare* plants in the mesocosms.

Light brown apple moth, *Epiphyas postvittana* (Walker):

Light brown apple moths were obtained from a colony maintained at Plant and Food Research in Auckland, New Zealand. Four 3rd instar caterpillars were added to the center of each +Herbivore mesocosm on 27 April 2017. A second introduction of three more caterpillars (of varying instar) was made on 6 June 2017 to supplement the low success of the first introduction. Due to the low success of both caterpillar introductions, two pairs of mated adult leafrollers were added during 17-23 October 2017. Moths were sent to Lincoln University as pupae and reared in containers, before pairs were moved to plastic cups, left to mate overnight, and then added to mesocosms. Surveys were conducted by systematically searching plants for caterpillars or the characteristic damage associated with leafrollers (i.e., webbing and rolling). Leaf rolls were gently examined and caterpillars of each leafroller species identified based on a combination of characters (i.e., size, head capsule color, and body color and patterning). Based on the Plant-SyNZ Database, light brown apple moths have been recorded feeding on 63 host plant species in New Zealand, and were recorded on 31 plant species in the mesocosms.

Brown-headed leafroller, *Ctenopseustis obliquana* (Walker):

Brown-headed leafrollers were also obtained from a colony maintained at Plant and Food Research in Auckland, New Zealand, and were introduced and surveyed using similar methodology to the other leafroller species. Four 3rd instar caterpillars were added to the center of each +Herbivore mesocosm on 28 April 2017. Due to the low survival of introduced caterpillars, two pairs of mated adult leafrollers were added during 17-22 October 2017. Based on the Plant-SyNZ Database, brown-headed leafroller have been recorded feeding on 140 host plant species in New Zealand, and were recorded on 23 plant species in the mesocosms.

Green-headed leafroller, *Planotortrix excessana* (Walker):

Green-headed leafrollers were also obtained from a colony maintained at Plant and Food Research in Auckland, New Zealand, and were introduced and surveyed using similar methodology to the other leafroller species. Four 3rd instar caterpillars were added to the center of each +Herbivore mesocosm on 28 April 2017. A second introduction of two more caterpillars (of varying instar) was made on 6 June 2017 to supplement the low success of the first introduction. Due to the low success of both caterpillar introductions, two pairs of mated adult leafrollers were added during 17-18 October 2017. Based on the Plant-SyNZ Database, green-headed leafroller have been recorded feeding on 35 host plant species in New Zealand, and were recorded on 21 plant species in the mesocosms.

Echium leaf miner, *Dialectica scalariella* (Zeller):

Echium leaf miner is an exotic biological control agent that attacks multiple species in the plant family Boraginaceae, and has been recorded feeding on 4 host plant species in New Zealand based on the Plant-SyNZ Database. This species was collected as leaf mines in various stages of development (from larvae to pupae) from *Echium vulgare* plants at Balmoral Lookout in Hurunui, New Zealand (42°52' S, 172°46' E). Leaf mines were placed in rearing cages and two adults (one of each sex) were added to each +Herbivore mesocosm between 7-17 June 2017. Pairs were collected from rearing cages during copulation to ensure females were mated. Plants were surveyed by carefully searching *Echium vulgare* for leaf mines. However, this species established in just a single mesocosm, where it was recorded on the expected host, *Echium vulgare*.

Greasy cutworm, *Agrotis ipsilon* (Hufnagel):

Greasy cutworm is a cosmopolitan caterpillar that self-colonized five mesocosms in low abundance. It was removed from -Herbivore mesocosms when encountered and left when found in +Herbivore mesocosms. Based on the Plant-SyNZ Database, greasy cutworms have been recorded feeding on 15 host plant species in New Zealand, and were recorded on 3 plant species in the mesocosms.

Common forest looper, *Pseudocoremia suavis* Butler:

Common forest looper is a native moth species that self-colonized five mesocosms. It was removed from -Herbivore mesocosms when encountered and left when found in +Herbivore mesocosms. Based on the Plant-SyNZ Database, common forest looper have been recorded feeding on 69 host plant species in New Zealand, and were recorded on 10 plant species in the mesocosms.

Leafhopper, *Anzygina zealandica* (Myers):

Anzygina zealandica is a common native leafhopper that self-colonized several mesocosms in high abundance and was subsequently identified using Knight (1976)[38]. We then added eight individuals of this species to each of the uncolonized +Herbivore mesocosms, collected using a sweep net from a mixture of grass species neighboring the experimental site. Plants were surveyed by systematically searching for leafhoppers, which were usually first noticed as they flew off the focal plant as it was being searched. If the host plant could not be positively identified (i.e., the leafhopper originated from a mixture of species or was on the herbivore cage) then the individual was ignored. Based on the Plant-SyNZ Database, *A. zealandica* have been recorded feeding on 13 host plant species in New Zealand, and were recorded on 37 plant species in the mesocosms.

Meadow spittlebug, *Philaenus spumarius* (L.):

Meadow spittlebug nymphs of varying instar were collected from a range of host plants at Balmoral Lookout, Hurunui on 12 November 2017, and Birdlings Flat, Banks Peninsula (43°48' S, 172°41' E), on 21 November 2017. Five nymphs were added to the center of each +Herbivore mesocosm on 13 November 2017, and an additional ten nymphs were added on 21 November 2017. Plants were surveyed by systematically searching for the characteristic spittle of nymphs or by observing adults feeding on plants during surveys. Based on the Plant-SyNZ Database, meadow spittlebugs have been recorded feeding on 17 host plant species in New Zealand, and were recorded on 30 plant species in the mesocosms.

Pea aphid, *Acyrthosiphon pisum* Harris:

Pea aphids self-colonized seven mesocosms where they fed on three exotic legume species. This herbivore species was removed from -Herbivore mesocosms when encountered and left when found in +Herbivore mesocosms. Plants were surveyed by systematically examining plant tissue for aphid colonies. If less than 500 aphids were found (as was always the case for this species), they were counted as accurately as possible. Based on the Plant-SyNZ Database, pea aphids have been recorded feeding on 11 host plant species in New Zealand, and were recorded on 3 plant species in the mesocosms.

Cherry-oat aphid, *Rhopalosiphum padi* (L.):

Cherry-oat aphids self-colonized several mesocosms, with a small number of plants experiencing severe outbreaks. Thus, we located a source population on Yorkshire fog-grass (*Holcus lanatus*) adjacent to the experimental site, which was used to infest previously uncolonized +Herbivore mesocosms with five alates (winged adults) on 27 October 2017. This herbivore species was also actively removed from -Herbivore mesocosms when

encountered. Plants were surveyed by systematically examining plant tissue for aphid colonies. If less than 500 aphids were found, these were counted as accurately as possible. For plants with larger colonies of aphids, we estimated aphid abundance to the nearest 10 (or nearest 100 for plants with over 2000 individuals). Based on the Plant-SyNZ Database, cherry-oat aphids have been recorded feeding on 35 host plant species in New Zealand, and were recorded on 9 plant species in the mesocosms.

Green peach aphid, *Myzus persicae* (Sulzer):

Green peach aphids were collected from *Rumex obtusifolius* adjacent to the experimental site and three alates (winged adults) were added to +Herbivore mesocosms on 21 November 2017. This herbivore species self-colonized several other mesocosms and was actively removed from -Herbivore mesocosms when encountered. Plants were surveyed by systematically examining plant tissue for aphid colonies. If less than 500 aphids were found, these were counted as accurately as possible. For plants with larger colonies of aphids, we estimated aphid abundance to the nearest 10. Based on the Plant-SyNZ Database, green peach aphids have been recorded feeding on 50 host plant species in New Zealand, and were recorded on 16 plant species in the mesocosms.

Foxglove aphid, *Aulacorthum solani* (Kaltenbach):

Foxglove aphids were collected from a field of lucerne (*Medicago sativa*) adjacent to the experimental site and five alates (winged adults) were added to +Herbivore mesocosms on 27 November 2017. This herbivore species self-colonized several other mesocosms and was actively removed from -Herbivore mesocosms when encountered. Plants were surveyed by systematically examining plant tissue for aphid colonies. If less than 500 aphids were found, these were counted as accurately as possible. For plants with larger colonies of aphids,

we estimated aphid abundance to the nearest 10. Based on the Plant-SyNZ Database, foxglove aphids have been recorded feeding on 81 host plant species in New Zealand, and were recorded on 7 plant species in the mesocosms.

Alpine grasshopper, *Paprides nitidus* Hutton:

Alpine grasshoppers were collected from Molesworth Station in North Canterbury, New Zealand (42°27' S, 172°49' E). An adult male and female pair were first added to the center of each +Herbivore mesocosm during 8-30 May 2017. Replacement additions occurred after each herbivore survey when either grasshoppers were not observed or were found deceased. Because the grasshoppers were highly mobile and host plant range could not always be reliably identified from direct observation, we used molecular analysis of DNA from grasshopper regurgitate and frass samples to accurately determine their host plants (see below for sample collection and molecular analysis protocol). Based on the Plant-SyNZ Database, *Paprides nitidus* grasshoppers have been recorded feeding on 72 host plant species in New Zealand, and were recorded on 38 plant species in the mesocosms.

Clover flea, *Sminthurus viridis* (L.):

Clover flea is an introduced springtail species from Europe with a broad host range but a preference for legumes. This species self-colonized 58% of mesocosm communities, but always in low abundance. It was removed from -Herbivore mesocosms when encountered and left when found in +Herbivore mesocosms. Plants were surveyed by systematically searching for clover fleas, which were usually first noticed as they hopped off the plant as it was being searched. If the host plant could not be positively identified (i.e., the clover flea was found on the mesocosm soil or herbivore cage) then the individual was ignored. Based on the Plant-SyNZ Database, clover fleas have been recorded feeding on 2 host plant species

in New Zealand (both *Trifolium* spp.), and were recorded on 32 plant species in the mesocosms.

Garden slug, *Deroceras* sp.:

Garden slugs self-colonized 71% of mesocosms. Slugs were removed from -Herbivore mesocosms when encountered and left when found in +Herbivore mesocosms. Surveys were conducted by systematically searching plants for slugs. Based on the Plant-SyNZ Database, *Deroceras* spp. slugs have been recorded feeding on 23 host plant species in New Zealand, and were recorded on 31 plant species in the mesocosms.

Black field cricket, *Teleogryllus commodus* (Walker):

Black field crickets were purchased from Inzect Direct (Wairarapa, New Zealand) and a single pair of male and female adults were introduced to +Herbivore mesocosms on 27 October 2017. However, this species failed to establish due to a late frost soon after their introduction.

Porina moth, *Wiseana copularis* (Meyrick):

Porina moth eggs were obtained from adult moths collected from pasture near Invermay, New Zealand (45°50' S, 170°22' E) in January 2017, and were stored at 4°C until the experiment was set up. We scattered 0.02 g of eggs (~250 eggs) throughout each +Herbivore mesocosm on 28 April 2017. However, we had no success in establishing this herbivore species.

Herbivore molecular diet analyses

For several highly mobile or belowground herbivore species, it was difficult or impossible to reliably characterize feeding interactions through direct observation. For these species, we used restriction fragment length polymorphism (RFLP) to identify host plants, using DNA extracted from frass, regurgitate, or gut contents. RFLP was considered well suited as a technique, given the low diversity of potential host plants (maximum of eight species, all of known identity), and allowed rapid and inexpensive identification of host plants. These molecular data were treated as any other observed plant-herbivore interaction, with the number of herbivore individuals observed to contain DNA of a given plant species weighted by mean biomass per individual and incorporated into the calculation of cumulative herbivore biomass for each individual plant and mesocosm. RFLP uses restriction enzymes to cut amplified DNA at enzyme-specific cutting sites and produce different sized DNA fragments that can be used to distinguish among genotypes, species, or broader taxonomic groups. By incubating samples overnight with up to three different restriction enzymes, we produced DNA fragment size combinations unique to the eight species in each of the 20 mesocosm communities, which were then visualized on agarose gel and cross-referenced against a database of known samples.

Sample collection and storage protocol:

Grasshoppers (*Paprides nitidus*) were non-destructively sampled by collecting regurgitate and frass samples during each herbivore survey. Regurgitate was collected by catching and handling grasshoppers, which frequently produce a small bubble of regurgitate as a defensive response to being handled, and this regurgitate could be collected into 1.7 mL Eppendorf tubes by holding their mouthparts to the lip of the tube. However, not all grasshoppers produced regurgitate and so on some occasions a sample could not be collected. All grasshoppers were then housed in clean rearing cups until they produced frass, which was

collected into Eppendorf tubes using sterile forceps, before the grasshopper was returned to its mesocosm. For grass grubs, we collected larvae into 1.7 mL Eppendorf tubes during the final mesocosm harvest by thoroughly searching plant roots and mesocosm soil. Fuller's rose weevil and clover root weevil larvae were sampled using the same approach as grass grubs and distinguished based on size and host plant associations. All samples were stored in a -80°C freezer until DNA extraction.

DNA extraction protocol:

Field-collected samples were used to first optimize DNA extraction, PCR, and RFLP protocols for processing of mesocosm grass grub and grasshopper samples. These optimized protocols are presented here. DNA was extracted from grass grub and weevil larvae samples using Qiagen DNEasy PowerSoil kits. Frozen samples were first surface sterilized to remove soil, external plant DNA, and other potential contaminants (10 seconds in distilled water, 30 seconds in 1% sodium hypochlorite, and two 10 second rinses in distilled water). The hindgut (fermentation sac) of grass grubs was then dissected from the rest of the body using sterile forceps, added to a 96-well plate on ice, and crushed with a sterile pestle. Whole weevil larvae were added to wells and crushed with a sterile pestle. Once the plate was full of samples, extraction was conducted following kit instructions.

DNA was extracted from grasshopper frass and regurgitate in their individual sample tubes using Sigma-Aldrich REDExtract-N-Amp kits. Before extraction, regurgitate samples were thawed and centrifuged (30 seconds at 10,000 rpm) to move the regurgitate sample from the lip of the tube to the bottom. Extraction was conducted by first adding 100 µL of extraction solution to each sample. At this point, a sterile pestle was used to crush frass samples, which were also vortexed and centrifuged (30 seconds at 10,000 rpm). Samples were then heated for 10 minutes at 95°C. Once cool, 300 µL of dilution solution was added to

each sample, which were vortexed and centrifuged (1 minute at 10,000 rpm). To reduce PCR inhibition, grasshopper frass and regurgitate samples were further diluted by 1:10 using dilution solution.

Polymerase chain reaction (PCR) protocol:

The primers used for all PCRs were ITS2F (5'-ATGCGATACTTGGTGTGAAT-3') and ITS3R (5'-GACGCTTCTCCAGACTACAAT-3')[39]. Each reaction for grass grub and weevil larvae samples used 2 µL of buffer, 0.4 µL of dNTPs, 1 µL of each primer (10 µM), 0.5 µL of bovine serum albumin (BSA), 0.16 µL of Taq polymerase, 1 µL of template DNA, and 13.94 µL of PCR grade water, for a total reaction volume of 20 µL. Reactions for grasshopper samples used 11 µL of REDExtract-N-Amp PCR mix, 2 µL of each primer (10 µM), 0.5 µL of template DNA, and 4.5 µL of PCR grade water, for a total reaction volume of 20 µL. All PCRs were run on Applied Biosystems Veriti PCR machines with a 5 minute ramp up period to 94°C, forty cycles of 30 s at 94°C, 30 s at 56°C and 45 s at 72°C, followed by an annealing period of 10 minutes at 72°C, before final cooling to 4°C. Negative and positive controls (*Lolium perenne* DNA) were included in each PCR to test for contamination or PCR failure. Successful DNA amplification was confirmed with gel electrophoresis on 1% agarose with RedSafe™ dye (Sigma-Aldrich) in 0.5X TBE buffer for 30 minutes at 100 V and 500 mA. We loaded 3 µL of PCR product and used 3 µL of Hyperladder 1kb to estimate amplicon size. Gels were visually examined using a Uvidoc HD2 UV photo machine.

Restriction fragment length polymorphism (RFLP) protocol:

We used the restriction enzymes TaqI, HaeIII, and MluCI, obtained from New England BioLabs (Ipswich, Massachusetts, United States) and selected based on virtual digestions using Sanger sequences for each mesocosm plant species. TaqI reactions used 2

µL of 1X CutSmart® Buffer, 0.5 µL of restriction enzyme, 8 µL of PCR product, and 9.5 µL of PCR grade water, for a total reaction volume of 20 µL. HaeIII and MluCI reactions used 2 µL of 1X CutSmart® Buffer, 1 µL of restriction enzyme, 8 µL of PCR product, and 9 µL of PCR grade water, for a total reaction volume of 20 µL. Samples were incubated overnight at 65°C for TaqI and 37°C for HaeIII and MluCI. The resulting fragments were visualized on 2.5% Invitrogen™ UltraPure™ low melting point agarose with RedSafe™ dye in 0.5X TBE buffer for 70 minutes at 100 V and 500 mA. We loaded 8 µL of digestion product mixed with 2 µL of 6X purple gel loading dye (New England BioLabs) into each lane.

Gels were visualized using the Uvidoc HD2 UV photo machine and band size was estimated by comparison to 5 µL of low molecular weight DNA ladder (25bp to 766 bp, New England BioLabs). Patterns of band sizes were cross-referenced against our database of real and virtual digests of Sanger sequences for each plant species to identify the host plant (or plants) in the sample. When the same host plant was identified from both regurgitate and frass samples collected during the same survey, duplicate results were removed from analyses. A subset of samples was also Sanger sequenced to confirm RFLP identification or to deal with any ambiguities in identification based on DNA fragment sizes.

Supplementary Notes

Trait-based analyses

To investigate potential mechanisms underlying invertebrate herbivore presence, diversity, biomass, and damage to plants, we explored whether variation in these response variables could be explained by the main effects and interactions between plant provenance and several traits of plants and herbivores. Traits examined included plant nitrogen fixing status (N-fixer or not), mycorrhizal association (ectomycorrhiza, arbuscular mycorrhiza, non-mycorrhizal), functional group (grass, forb, shrub, or tree), specific leaf area (SLA), total plant biomass, and insect reproduction status (whether or not the herbivore species produced multiple generations in the mesocosms, as indicated by the presence of younger life stages or larger abundance than the initial introduction). SLA is a plant trait indicative of growth strategy, where high values are associated with fast growth rates[40], high palatability[41], and successful invasive species[42]. SLA for our experimental plant species was obtained from Waller et al. (2020)[43]. Insect reproduction status represents a trait outcome that allowed us to distinguish between aggregative vs. population responses of herbivores. We predicted that plant-herbivore interactions would be strongest on nitrogen-fixing and mycorrhizal plants, grasses and forbs, plants with high SLA and biomass, and for herbivore species that reproduced in the mescososms. To test these predictions, we modelled each response variable (transformations and model error distributions were the same as in Supplementary Table 26) as a function of the trait main effects and their pairwise interactions with plant provenance, and with the random effects of plant species, herbivore species (for models of herbivore presence and biomass only), and mesocosm nested within plant community.

We found that the probability of an invertebrate herbivore species occurring on a given plant was 2.3 times higher on plants that do not fix nitrogen than on nitrogen-fixers ($F = 15.29$, $P =$

0.002; Supplementary Table 19), and increased with total plant biomass (F = 24.95, P = 0.00003; Supplementary Table 19). Moreover, the probability of a herbivore species occurring on a given plant also depended on the interaction between plant provenance and insect reproduction status in the mesocosms (F = 6.00, P = 0.015; Supplementary Fig. 6, Supplementary Table 19). No pairwise post-hoc Tukey tests (Bonferroni corrected) were statistically significant, although main effects showed that herbivore species were more likely to interact with exotic than native plants (F = 35.75, P = 0.0003; Supplementary Table 19). Herbivore species biomass on plants also depended on the interaction between plant provenance and insect reproduction status ($F_{1,1406}$ = 66.76, P = 6.8e^{-16}; Supplementary Fig. 7, Supplementary Table 20). Again, none of the pairwise post-hoc contrasts were statistically significant, but herbivore biomass was 5.3 times higher for species that did not reproduce in the mesocosms (insect reproduction status main effect: $F_{1,18}$ = 4.88, P = 0.040; Supplementary Table 20), likely driven by the high biomass of non-reproductive species such as the alpine grasshopper *Paprides nitidus*. Furthermore, the relationship between herbivore species biomass and plant biomass differed beteween native and exotic plants (plant biomass × plant provenance interaction: $F_{1,382}$ = 4.10, P = 0.044; Supplementary Fig. 8, Supplementary Table 20), with a positive relationship observed for native plants (slope = 0.0007, t = 3.47, P = 0.001) and no relationship for exotics (slope = 0.0002, t = 1.05, P = 0.295). Herbivore normalized degree (i.e., the proportion of herbivore species that fed on a given host plant out of the total herbivore species in the mesocosm) increased strongly with total biomass of exotic plants (slope = 0.0003, t = 4.84, P = 0.000002, n = 259) but had no significant relationship with total biomass of native plants (slope = 0.0001, t = 1.78, P = 0.076, n = 262; plant biomass × plant provenance interaction: $F_{1,348}$ = 5.01, P = 0.026; Supplementary Fig. 9, Supplementary Table 21). Herbivore chewing and scraping damage was 38% higher on exotic plants that do not fix nitrogen compared with nitrogen-fixers (P =

0.022), although the same effect was not observed for native plants (nitrogen fixing status × plant provenance interaction: $F = 8.96$, $P = 0.043$; Supplementary Fig. 10, Supplementary Table 22). Finally, compared with plants that associate with ectomycorrhizal fungi, herbivore chewing and scraping damage was 69% ($P = 0.002$) and 56% ($P = 0.007$) higher for non-mycorrhizal plants and plants that associate with arbuscular mycorrhizal fungi, respectively ($F = 25.90$, $P = 0.003$; Supplementary Fig. 11, Supplementary Table 22).

Supplementary References

[1]Meijer, K., Schilthuizen, M., Beukeboom, L. & Smit, C. A review and meta-analysis of the enemy release hypothesis in plant-herbivorous insect systems. *PeerJ* **4**, e2560v1 (2016).

[2]Agrawal, A.A. & Kotanen, P.M. Herbivores and the success of exotic plants: a phylogenetically controlled experiment. *Ecol. Lett.* **6**, 712–715 (2003).

[3]Agrawal, A.A. et al. Enemy release? An experiment with congeneric plant pairs and diverse above- and belowground enemies. *Ecology* **86**, 2979-2989 (2005).

[4]Auerbach, M. & Simberloff, D. Rapid leaf-miner colonization of introduced trees and shifts in sources of herbivore mortality. *Oikos* **52**, 41-50 (1988).

[5]Bürki, C. & Nentwig, W. Comparison of herbivore insect communities of *Heracleum sphondylium* and *H. mantegazzianum* in Switzerland (Spermatophyta: Apiaceae). *Entomol. Gen.* **22**, 147-155 (1997).

[6]Carpenter, D. & Cappuccino, N. Herbivory, time since introduction and the invasiveness of exotic plants. *J. Ecol.* **93**, 315-321 (2005).

[7]Cincotta, C.L., Adams, J.M. & Holzapfel, C. Testing the enemy release hypothesis: a comparison of foliar insect herbivory of the exotic Norway maple (*Acer platanoides* L.) and the native sugar maple (*A. saccharum* L.). *Biol. Invasions* **11**, 379-388 (2008).

[8]Engelkes, T., Wouters, B., Bezemer, T.M., Harvey, J.A. & van der Putten, W.H. Contrasting patterns of herbivore and predator pressure on invasive and native plants. *Basic Appl. Ecol.* **13**, 725-734 (2012).

[9]Goßner, M., Liston, A. & Späth, J. Sawflies in the crowns of native and exotic trees, sampled with flight-interception traps in southern Germany (Hymenoptera: Symphyta). *Entomol. Gen.* **30**, 273-282 (2007).

[10]Hartley, M.K., Rogers, W.E. & Siemann, E. Comparisons of arthropod assemblages on an invasive and native trees: abundance, diversity and damage. *Arthropod Plant Interact.* **4**, 237-245 (2010).

[11]Harvey, K.J., Nipperess, D.A., Britton, D.R. & Hughes, L. Comparison of invertebrate herbivores on native and non-native *Senecio* species: implications for the enemy release hypothesis. *Austral Ecol.* **40**, 503-514 (2015).

[12]Heard, M.J. & Sax, D.F. Coexistence between native and exotic species is facilitated by asymmetries in competitive ability and susceptibility to herbivores. *Ecol. Lett.* **16**, 206-213 (2013).

[13]Helden, A.J., Stamp, G.C. & Leather, S.R. Urban biodiversity: comparison of insect assemblages on native and non-native trees. *Urban Ecosyst.* **15**, 611-624 (2012).

[14]Hill, S.B. & Kotanen, P.M. Phylogenetically structured damage to Asteraceae: susceptibility of native and exotic species to foliar herbivores. *Biol. Invasions* **12**, 3333-3342 (2010).

[15]Jobin, A., Schaffner, U. & Nentwig, W. The structure of the phytophagous insect fauna on the introduced weed *Solidago altissima* in Switzerland. *Entomol. Exp. Appl.* **79**, 33-42 (1996).

[16]Kennedy, C.E.J. & Southwood, T.R.E. The number of species of insects associated with British trees: a re-analysis. *J. Anim. Ecol.* **53**, 455-478 (1984).

[17]Leather, S.R. Insect species richness of the British Rosaceae: the importance of host range, plant architecture, age of establishment, taxonomic isolation and species-area relationships. *J. Anim. Ecol.* **55**, 841-860 (1986).

[18]Lieurance, D. & Cipollini, D. Exotic *Lonicera* species both escape and resist specialist and generalist herbivores in the introduced range in North America. *Biol. Invasions* **15**, 1713-1724 (2013).

[19]Liu, H. & Stiling, P. Testing the enemy release hypothesis: a review and meta-analysis. *Biol. Invasions* **8**, 1535-1545 (2006).

[20]Liu, H., Stiling, P. & Pemberton, R.W. Does enemy release matter for invasive plants? Evidence from a comparison of insect herbivore damage among invasive, non-invasive and native congeners. *Biol. Invasions* **9**, 773-781 (2007).

[21]Lombardero, M.J., Vázquez-Mejuto, P. & Ayres, M.P. Role of plant enemies in the forestry of indigenous vs. non indigenous pines. *Ecol. Appl.* **18**, 1171-1181 (2008).

[22]Meijer, K. et al. Phytophagous insects on native and non-native host plants: combining the community approach and the biogeographical approach. *PLoS ONE* **10**, e0125607 (2015).

[23]Novotny, V. et al. Colonising aliens: caterpillars (Lepidoptera) feeding on *Piper aduncum* and *P. umbellatum* in rainforests of Papua New Guinea. *Ecol. Entomol.* **28**, 704-716 (2003).

[24]Procheş, S., Wilson, J.R.U., Richardson, D.M. & Chown, S.L. Herbivores, but not other insects, are scarce on alien plants. *Austral Ecol.* **33**, 691-700 (2008).

[25]Radho-Toly, S., Majer, J.D. & Yates, C. Impact of fire on leaf nutrients, arthropod fauna and herbivory of native and exotic eucalypts in Kings Park, Perth, Western Australia. *Austral Ecol.* **26**, 500-506 (2001).

[26]Schutzenhofer, M.R., Valone, T.J. & Knight, T.M. Herbivory and population dynamics of invasive and native *Lespedeza*. *Oecologia* **161**, 57-66 (2009).

[27]Southwood, T.R.E., Moran, V.C. & Kennedy, C.E.J. The richness, abundance and biomass of the arthropod communities on trees. *J. Anim. Ecol.* **51**, 635-649 (1982).

[28]Southwood, T.R.E., Wint, G.R.W., Kennedy, C.E.J. & Greenwood, S.R. Seasonality, abundance, species richness and specificity of the phytophagous guild of insects on oak (*Quercus*) canopies. *Eur. J. Entomol.* **101**, 43-50 (2004).

[29]Sugiura, S. Associations of leaf miners and leaf gallers with island plants of different residency histories. *J. Biogeogr.* **37**, 237-244 (2010).

[30]Yela, J.L. & Lawton, J.H. Insect herbivore loads on native and introduced plants: a preliminary study. *Entomol. Exp. Appl.* **85**, 275-279 (1997).

[31]Zuefle, M.E., Brown, W.P. & Tallamy, D.W. Effects of non-native plants on the native insect community of Delaware. *Biol. Invasions* **10**, 1159-1169 (2008).

[32]Ghanizadeh, H. & Harrington, K.C. Weed management in New Zealand pastures. *Agronomy* **9**, 448 (2019).

[33]Howell, C. *Consolidated list of environmental weeds in New Zealand*. DOC Research & Development Series 292 (Department of Conservation, Wellington, New Zealand, 2008).

[34]Kelsey, J.M. Note on morphological differences between sexes of adult *Costelytra zealandica* (White). *New Zeal. J. Sci.* **8**, 173 (1965).

[35]Kain, W.M. Identification of the sexes in *Costelytra zealandica* (Coleoptera: Scarabaeidae). *New Zeal. J. Sci.* **15**, 85–87 (1972).

[36]Bright, D.E. Revision of the genus *Sitona* (Coleoptera: Curculionidae) of North America. *Ann. Entomol. Soc. Am.* **87**, 277–306 (1994).

[37]Velázquez De Castro, A.J., Alonso-Zarazaga, M.A. & Outerelo, R. Systematics of Sitonini (Coleoptera: Curculionidae: Entiminae), with a hypothesis on the evolution of feeding habits. *Syst. Entomol.* **32**, 312-331 (2007).

[38]Knight, W.J. Typhlocybinae of New Zealand (Homoptera: Cicadellidae). *New Zeal. J. Zool.* **3**, 71-87 (1976).

[39]Chen, S.L. et al. Validation of the ITS2 region as a novel DNA barcode for identifying medicinal plant species. *PLoS One* **5**, e8613 (2010).

[40]Wright, I.J. et al. The worldwide leaf economics spectrum. *Nature* **428**, 821 (2004).

[41]Pérez-Harguindeguy, N. et al. Leaf traits and herbivore selection in the field and in cafeteria experiments. *Austral Ecol.* **28**, 642-650 (2003).

[42]van Kleunen, M., Weber, E. & Fischer, M. A meta-analysis of trait differences between invasive and non-invasive plant species. *Ecol. Lett.* **13**, 235–245 (2010).

[43]Waller, L.P. et al. Biotic interactions drive ecosystem responses to plant invaders. *Science* **368**, 967-972 (2020).
