# Supplementary material for: Exotic plants accumulate and share herbivores yet dominate communities via rapid growth
Source: Nat Commun. 2021 May 11;12:2696. doi: 10.1038/s41467-021-23030-1 (PMC8113582; doi:10.1038/s41467-021-23030-1)
Supplement: Supplementary file 3 — Reporting Summary [file 41467_2021_23030_MOESM3_ESM.pdf]

nature research

Corresponding author(s): Warwick Allen

Last updated by author(s): Mar 30, 2021

Reporting Summary

Nature Research wishes to improve the reproducibility of the work that we publish. This form provides structure for consistency and transparency in reporting. For further information on Nature Research policies, see our Editorial Policies and the Editorial Policy Checklist.

Statistics

For all statistical analyses, confirm that the following items are present in the figure legend, table legend, main text, or Methods section.

n/a	Confirmed	
☐	☒	The exact sample size (*n*) for each experimental group/condition, given as a discrete number and unit of measurement
☐	☒	A statement on whether measurements were taken from distinct samples or whether the same sample was measured repeatedly
☐	☒	The statistical test(s) used AND whether they are one- or two-sided<br>*Only common tests should be described solely by name; describe more complex techniques in the Methods section.*
☐	☒	A description of all covariates tested
☐	☒	A description of any assumptions or corrections, such as tests of normality and adjustment for multiple comparisons
☐	☒	A full description of the statistical parameters including central tendency (e.g. means) or other basic estimates (e.g. regression coefficient) AND variation (e.g. standard deviation) or associated estimates of uncertainty (e.g. confidence intervals)
☐	☒	For null hypothesis testing, the test statistic (e.g. F, t, r) with confidence intervals, effect sizes, degrees of freedom and P value noted<br>*Give P values as exact values whenever suitable.*
☒	☐	For Bayesian analysis, information on the choice of priors and Markov chain Monte Carlo settings
☐	☒	For hierarchical and complex designs, identification of the appropriate level for tests and full reporting of outcomes
☒	☐	Estimates of effect sizes (e.g. Cohen's *d*, Pearson's *r*), indicating how they were calculated

Our web collection on statistics for biologists contains articles on many of the points above.

Software and code

Policy information about availability of computer code

Data collection	No software used for data collection in this study.
Data analysis	All analyses were performed in R 3.6.1 using the lme4 (version 1.1-21), emmeans (version 1.3.5.1), and bipartite (version 2.13) packages.

For manuscripts utilizing custom algorithms or software that are central to the research but not yet described in published literature, software must be made available to editors and reviewers. We strongly encourage code deposition in a community repository (e.g. GitHub). See the Nature Research guidelines for submitting code & software for further information.

Data

Policy information about availability of data

All manuscripts must include a data availability statement. This statement should provide the following information, where applicable:

- Accession codes, unique identifiers, or web links for publicly available datasets
- A list of figures that have associated raw data
- A description of any restrictions on data availability

Data available for download from Dryad: https://doi.org/10.5061/dryad.0vt4b8gzd.

Field-specific reporting

Ecological, evolutionary & environmental sciences study design

All studies must disclose on these points even when the disclosure is negative.

Study description	We established 160 experimental mesocosm communities, where interactions between plants, invertebrate herbivores and soil biota were manipulated and measured. Mesocosms were planted with one of 20 unique communities, each consisting of eight plant species (Table S2.1) selected from a pool of 39 plant species that co-occur in New Zealand grassland communities (19 natives, 20 exotics, Table S2.2). Plant communities were designed to vary orthogonally in their proportion of exotic and woody species (0-100% and 0-63%, respectively, Fig. 1B). Each plant community was replicated eight times to allow the duplicate application of herbivore and soil treatments (described below), with plant communities blocked together to minimize any environmental gradients.<br><br>We manipulated invertebrate herbivores across mesocosm communities (Fig. 1B). All mesocosms were covered with large mesh cages and herbivore populations were deliberately established in 80 mesocosms. Thirteen herbivore species that were added successfully established, along with seven self-colonizing species, totaling 20 different species (establishment success and other herbivore species characteristics are detailed in Table S2.3). These species were mostly polyphagous (four oligophagous species established, but all in low abundance; Table S2.3) and included 7 native and 13 exotic herbivores from multiple feeding guilds (leaf and root chewers, suckers, and miners). Each herbivore species was added to all +Herbivore mesocosms in equal density, regardless of whether a known host plant was present. Herbivore additions were staggered depending upon availability and some species were added multiple times to increase probability of establishment success and maintain populations. All self-colonizing species were regularly removed from -Herbivore mesocosms, including spillover from intentional additions, but were allowed to establish populations in +Herbivore mesocosms.<br><br>The herbivore treatment was crossed with a soil biota manipulation ('home' vs. 'away') (Fig. 1B). Soil biota was manipulated using a modified plant-soil feedback approach, where we grew each plant species in monoculture in 10 L pots of field-collected soil and pasteurized sand (50:50 mix) prior to the experiment to culture their associated soil biota. These conditioned soils were harvested after 9-10 months and used to create 'home' and 'away' soil inoculum mixtures for each plant community that were added to the mesocosms. 'Home' soils contained conditioned soils mixed from the eight species occurring in that community, whereas 'away' soils contained conditioned soils mixed from eight species occurring in one of the other 19 communities, but where a focal species did not occur.
Research sample	We used 39 plant species and 20 invertebrate herbivore species for the experiment (all species are named in Tables S2.2 and S2.3). These species were selected to be representative of communities that naturally occur in New Zealand grasslands.
Sampling strategy	The total number of mesocosms (n = 160) was selected to be as large as possible, while still remaining a feasible experiment that could be completed successfully. Each plant was thoroughly sampled for invertebrate herbivores on eight separate occasions throughout the experiment. This represented as many samples as was feasible, given the time constraints of the field work (it took up to three weeks at a time to sample all of the plants in the mesocosms).
Data collection	We measured herbivore richness, biomass, chew damage, and plant biomass (Fig. 1C). Herbivores were surveyed by Warwick Allen on eight occasions: May, June, July, August, September and November in 2017 and January and April in 2018. For each survey, we counted the number of individuals of each herbivore species that were observed feeding on each plant. For species that reached high densities (e.g., aphids), abundance was estimated by surveying a portion of the plant and extrapolating to the entire plant. For some highly mobile or belowground herbivores it was difficult to reliably characterize feeding interactions through direct observation. For these species, we used restriction fragment length polymorphism (RFLP) to identify host plants with DNA extracted from frass, regurgitate, or gut contents (see Appendix S2 for detailed description of molecular protocols). Finally, because we could not practically measure the biomass of each individual herbivore from each mesocosm, we converted raw abundances to a standardized estimate of herbivore biomass for each species using mean dry biomass of a random sample of 10 individuals. For each survey, we also assessed leaf damage of each plant against six different categories (0 = no damage, 1 = 1-5% leaf tissue chewed, 2 = 6-25%, 3 = 26-50%, 4 =51-75%, 5 = >75%). We used these categories because of the large number of plants to survey and the difficulties of non-destructively measuring percent leaf damage at finer resolution in situ. Finally, plants were harvested after one year, and total biomass washed, dried at 65°C, and weighed.
Timing and spatial scale	Plants were planted into mesocosm pots during March 2017 and grown for just over one year before being harvested in April 2018. Herbivores were surveyed on eight occasions: May, June, July, August, September and November in 2017 and January and April in 2018. These survey dates were used to collect data on herbivore abundance throughout the year.
Data exclusions	The herbivore exclusion treatment was highly successful in reducing herbivore richness, biomass, and damage across the experiment (Appendix S3, Fig. S3.1). Therefore, only data from the herbivore addition mesocosms were used to test our predictions, except for those relating to direct and indirect herbivore impacts on plant biomass (predictions 2c and 3a, b in Table 1; see statistical analyses below). Additionally, for analyses of herbivore presence and biomass, we retained absent interactions (i.e., zeroes in the data) that were within the fundamental host range for each herbivore species (based on the experiment-wide meta-web; i.e., the herbivore species fed on the focal host in at least one mesocosm) and discarded data for those that were not.
Reproducibility	Due to the large-scale nature of the experiment, we did not attempt to repeat it.
Randomization	Individual plants were randomly assigned to mesocosm pots, as were invertebrate herbivores.
Blinding	In the field, mesocosms were labeled with only their number and not their treatments. This meant that observers were unaware of whether herbivores were added or reduced until after they had completed their survey, or how the soil was manipulated ('home' or 'away').

Did the study involve field work? ☒ Yes ☐ No

Field work, collection and transport

Field conditions	We collected seeds, soil and invertebrate herbivores from the field for use in the mesocosm experiment, but not any data. Therefore, field conditions were not considered to be important to the outcome of the experiment.
Location	Seeds of the experimental plant species were collected from sites across the norther half of New Zealand's South Island, from Molesworth Station in the Marlborough region in the north, Arthur's Pass to the south and west, and Banks Peninsula to the south and east. Likewise, inoculum soil for use in the experiment was collected from 12 field sites in the same geographic area, chosen because subsets of our focal plant species were present. These sites included: Castle Hill (-43.20052, 171.72916); Cora Lynn, Wilderness Lodge (-43.02813, 171.66296); White Bridge, Hawdon Valley (-42.99166, 171.74894); Mt. Cheeseman (-43.18067, 171.69236); Mt. Cheeseman (-43.18057, 171.69287); Orton Bradley Park (-43.67289, 172.71307); Orton Bradley Park (-43.66658, 172.71002); Molesworth Station (-42.47243, 172.82729); Molesworth Station/Hanmer Springs (-42.54925, 172.82746); Brooksdale Station (-43.26112, 171.68406); Glenthorne (-43.20225, 171.44980); and Glenthorne Manuka (-43.16672, 171.41314).<br><br>Several invertebrate herbivore species were also collected from field sites within the same geographic area: On 30-31 May 2017, clover root weevils and lucerne weevils were collected using a vacuum sampler from patches of clover (Trifolium sp.) and a field of lucerne (Medicago sativa), respectively, that were adjacent to the experimental site (-43.646471, 172.452958). Echium leaf miner was collected as leaf mines in various stages of development (from larvae to pupae) from Echium vulgare plants at Balmoral Lookout in Hurunui, New Zealand (-42.874286, 172.770229). Meadow spittlebug nymphs of varying instar were collected from a range of host plants at Balmoral Lookout, Hurunui on 12 November 2017, and Birdlings Flat, Banks Peninsula (-43.815733, 172.700020), on 21 November 2017. Alpine grasshoppers were collected from Balmoral Lookout area in Hurunui, New Zealand (-42.874286, 172.770229). Adult grass grubs were collected at dusk on 10 and 23 November 2017 from a garden near Southbridge, New Zealand (-43.807831, 172.275742).
Access & import/export	We had permission to visit and obtain seed, soil, and invertebrate herbivores from all field locations, which were mostly owned by private landowners. Permission was granted by a diverse range of stakeholders, from private landowners through to the Department of Conservation (Authorisation Number 52457-RES). No importing or exporting of samples was required for the project.
Disturbance	Soil and invertebrate herbivores were taken from the field for use in the experiment. Soil disturbance was minimized by only taking soil from a small area (usually only around 2 x 2 m) and replanting the small number of plants that were disturbed by our digging. Impacts on the invertebrate herbivore populations that we sampled were considered to be minimal. This was because they were usually highly abundant species and we spread our sampling out across a large area.

Reporting for specific materials, systems and methods

We require information from authors about some types of materials, experimental systems and methods used in many studies. Here, indicate whether each material, system or method listed is relevant to your study. If you are not sure if a list item applies to your research, read the appropriate section before selecting a response.

Materials & experimental systems

n/a	Involved in the study	
x		Antibodies
x		Eukaryotic cell lines
x		Palaeontology and archaeology
	x	Animals and other organisms
x		Human research participants
x		Clinical data
x		Dual use research of concern

Methods

n/a	Involved in the study	
x		ChIP-seq
x		Flow cytometry
x		MRI-based neuroimaging

Animals and other organisms

Policy information about studies involving animals; ARRIVE guidelines recommended for reporting animal research

Laboratory animals	The study did not involve laboratory animals.
Wild animals	Several species of insect were collected from the field for use in the experiment. These were: New Zealand grass grub (Costelytra giveni), clover root weevil (Sitona obsoletus), lucerne weevil (Sitona discoideus), alpine grasshopper (Paprides nitidus), Echium leaf miner (Dialectica scalariella), and meadow spittlebug (Philaenus spumarius). Other insects were either obtained from colonies that were previously established at another institution (e.g., leafroller caterpillars from Plant and Food Research), or had colonized mesocosm communities naturally and were subsequently spread around the +Herbivore treatment mesocosms.<br><br>The insects were captured by locating them on a host plant and collecting them into an appropriate container. Clover root weevil and lucerne weevil were collected using a vacuum sampler. All insects were transported in a cooler to the mesocosm experiment to avoid them overheating in the car.<br><br>After the study, the invertebrate herbivores were released back into their natural populations, except for a subsample that were frozen and used to estimate mean biomass for each species.

Field-collected samples	Field-collected invertebrate herbivores were added to the mesocosm plant communities, where they were allowed to establish and feed on any of the possible host plants. At the end of the experiment, the invertebrate herbivores were released back into their natural populations, except for a subsample that were frozen and used to estimate mean biomass for each species.
Ethics oversight	No ethics approval was required, as we were working with invertebrate herbivores (i.e., insects and slugs), taxa that are not covered by ethics oversight in New Zealand.

Note that full information on the approval of the study protocol must also be provided in the manuscript.
